# Species divergence and environmental adaptation of *Picea asperata* complex at the whole genome level

**DOI:** 10.1002/ece3.70126

**Published:** 2024-08-06

**Authors:** Yifu Liu, Wenfa Xiao, Fude Wang, Ya Wang, Yao Dong, Wen Nie, Cancan Tan, Sanping An, Ermei Chang, Zeping Jiang, Junhui Wang, Zirui Jia

**Affiliations:** ^1^ Key Laboratory of Forest Ecology and Environment of National Forestry and Grassland Administration, Ecology and Nature Conservation Institute Chinese Academy of Forestry Beijing China; ^2^ State Key Laboratory of Tree Genetics and Breeding Chinese Academy of Forestry Beijing China; ^3^ Heilongjiang Forestry Research Institute Harbin China; ^4^ Key Laboratory of Tree Breeding and Cultivation of National Forestry and Grassland Administration, Research Institute of Forestry Chinese Academy of Forestry Beijing China; ^5^ Research Institute of Forestry of Xiaolong Mountain Gansu Provincial Key Laboratory of Secondary Forest Cultivation Tianshui China

**Keywords:** adaptive evolution, gene flow, genetic divergence, *Picea*, selective sweep

## Abstract

To study the interspecific differentiation characteristics of species originating from recent radiation, the genotyping‐by‐sequencing (GBS) technique was used to explore the kinship, population structure, gene flow, genetic variability, genotype–environment association and selective sweeps of *Picea asperata* complex with similar phenotypes from a genome‐wide perspective. The following results were obtained: 14 populations of *P. asperata* complex could be divided into 5 clades; *P. wilsonii* and *P. neoveitchii* diverged earlier and were more distantly related to the remaining 6 spruce species. Various geological events have promoted the species differentiation of *P. asperata* complex. There were four instances of gene flow among *P. koraiensis*, *P. meyeri*, *P. asperata*, *P. crassifolia* and *P. mongolica*. The population of *P. mongolica* had the highest level of nucleotide diversity, and *P. neoveitchii* may have experienced a bottleneck recently. Genotype–environment association found that a total of 20,808 genes were related to the environmental variables, which enhanced the adaptability of spruce in different environments. Genes that were selectively swept in the *P. asperata* complex were primarily associated with plant stress resistance. Among them were some genes involved in plant growth and development, heat stress, circadian rhythms and flowering. In addition to the commonly selected genes, different spruce species also displayed unique genes subjected to selective sweeps that improved their adaptability to different habitats. Understanding the interspecific gene flow and adaptive evolution of *Picea* species is beneficial to further understanding the species relationships of spruce and can provide a basis for studying spruce introgression and functional genomics.

## Graphical abstract

To understand the mechanisms of interspecific differentiation within *Picea*, we analysed the genetic relationships and environmental adaptability of eight phenotypically similar species of *Picea*. It was found that these eight species could be differentiated into five evolutionary branches. The estimated divergence times coincide with the estimated timing of mountain uplifts in their distribution areas. Selective sweep analysis revealed that the selected genes are primarily associated with plant stress resistance, morphogenesis and circadian rhythms. The results contribute to understanding the adaptive evolutionary patterns of *Picea* to ecological environments and provide a basis for the genetic improvement of *Picea*.

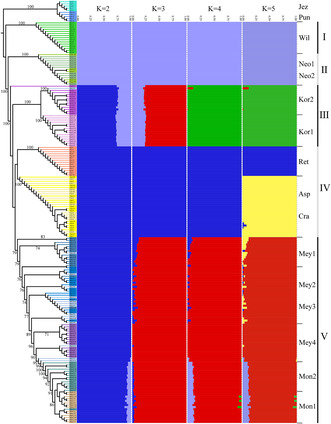

## 1 INTRODUCTION

*Picea* is the third biggest genus in the Pinaceae family, which is a key aspect of Northern Hemisphere boreal, montane and subalpine forests and has important ecological and economic value. There are approximately 38 species (Farjon, 2017), of which approximately 18 occur in China (Fu et al., 1999). *Picea* is widespread throughout the Northern Hemisphere, but the reproductive isolation within *Picea* is weak, and there is extensive gene flow and reticulate evolution among species (Shen et al., 2019), which causes convergence of phenotypic traits, and sometimes the conspecific populations distributed in remote mountain areas are considered to be different subspecies or varieties due to slight differences in morphology (Farjon, 2017). These factors make the evolutionary history, classification, and relationship between species of *Picea* very complicated. To better comprehend the evolutionary history of *Picea* and elucidate the classification of species within the genus, it is of utmost importance to comprehend the gene flow and selection pressure of interspecies differentiation among these morphologically similar spruce species.

Heterogeneity in the environment exerts strong selection pressure on species, in turn promoting the differentiation of species (Bian et al., 2020), and this evolution driven by natural selection is an important force supporting speciation (Charles, 1871). Therefore, understanding the mechanisms by which environmental selection pressures drive genetic differentiation is critical to studying species evolution. At present, most studies on selective sweeps during plant species differentiation focus on domesticated cash crops. Wu et al. (2019) performed selective sweep analysis on 991 accessions of *Brassica napus* and found genes related to the regulation of plant development and stress response. Other scholars have also detected selective sweeps in fruit trees such as *Macadamia integrifolia*, *Prunus salicina*, *Juglans regia* and *Malus* (Duan et al., 2017; Huang et al., 2021; Ji et al., 2021; Lin et al., 2022), where *M. integrifolia* genes are mainly related to fatty acid biosynthesis, seed coat development and heat stress response, and the selected genes in *J. regia* are mainly involved in adaptation to temperature, precipitation and altitude. The selected genes in *Prunus salicina* and *Malus* are mainly related to fruit quality or flavour, and the selected genes in *Prunus salicina* are also related to flowering and stress resistance. At present, there are few studies on the selective sweeps of natural spruce populations. The selective sweeps of two extremely related species, *P. asperata* and *P. crassifolia*, distributed on the Tibetan Plateau were analysed by Feng et al. (2023). The selected genes were predominantly associated with temperature and precipitation. In studies of *P. wilsonii*, *P. neoveitchii* and *P. likiangensis*, it was discovered that the majority of the selected genes were linked with plant stress resistance, flowering and morphogenesis (Liu, Qin, et al., 2022). At present, there are two primary explanations for the genesis of *Picea*: East Asia (Wright, 1955) and North America (Ran et al., 2015; Shao et al., 2019). Regardless of origin, northern China is an essential migration route for *Picea*, connecting with the Qinghai–Tibet Plateau, and the secondary distribution centre of *Picea*, *P. koraiensis*, *P. meyeri*, *P. mongolica*, *P. wilsonii*, *P. neoveitchii*, *P. asperata*, *P. crassifolia* and *P. retroflexa* are distributed in this area. Although these eight spruce species span a large distribution area, from northeast China to northwest China and the edge of the Qinghai–Tibet Plateau, they are closely related (Feng et al., 2019) and phenotypically similar, so we refer to these eight species as *P. asperata* complex. Due to interspecific hybridisation and frequent recombination of *Picea* to form chimeric plastids, it is difficult to unify phylogenetic relationships reconstructed from different plastids (Sullivan et al., 2017), a small number of nuclear genes contain limited genetic information and whole‐genome resequencing costs are prohibitive. Therefore, *P. pungens* and *P. jezoensis* were employed as outgroups in this investigation, and *P. asperata* complex was selected as materials, aiming to obtain genome‐wide single nucleotide polymorphism (SNP) markers by genotyping‐by‐sequencing (GBS) technology to solve the following three questions and provide scientific justification for the introduction and classification of *Picea*: (1) Is there gene flow among the *P. asperata* complex leading to close kinship and phenotypic similarity? (2) How did the *P. asperata* complex diverge due to geological changes? (3) Were the *P. asperata* complex subject to environmental selection pressures and how did they adapt to the differentiated environment?

## 2 MATERIALS AND METHODS

### 2.1 Sample collection

**FIGURE 1:**
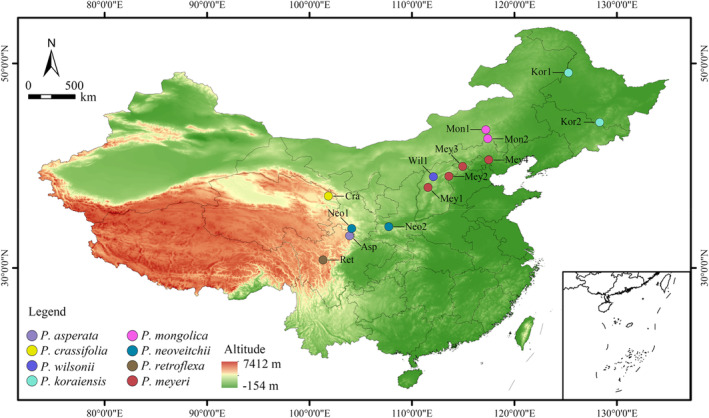
Location of 15 spruce populations sampled from *Picea asperata* complex. Wil: *P. wilsonii*; Neo1 and Neo2: *P. neoveitchii* from Gansu and Shaanxi, respectively; Kor1 and Kor2: *P. koraiensis* from Gaofeng and Hailing, respectively; Ret: *P. retroflexa*; Cra: *P. crassifolia*; Asp: *P. asperata*; Mey1, Mey2, Mey3 and Mey4: *P. meyeri* from Pangquangou, Mount Wutai, Xiaowutai Mountain and Wuling Mountain, respectively; Mon1 and Mon2: *P. mongolica* from Baiyinaobao and Huamugou respectively.

Needles were gathered from 14 different wild populations representing 8 different species of *Picea* for this investigation, and needle samples were obtained from a total of 190 individuals, including 60 individuals from 4 *P. meyeri* populations, 29 individuals from 2 *P. mongolica* populations, 28 individuals from 2 *P. koraiensis* populations, 15 individuals from 2 *P. neoveitchii* populations, 17 individuals from 1 *P. asperata* population, 15 individuals from 1 *P. wilsonii* population, 14 individuals from 1 *P. retroflexa* population and 12 individuals from 1 *P. crassifolia population*. We also collected needles from five individuals each of *P. jezoensis* and *P. pungens* to serve as outgroups. The sampled trees in each population were more than 100 m apart, and the collected needles were kept at a low temperature after being quickly dried using silica gel. Each population location was recorded together with its latitude, longitude and altitude using an eTrex portable GPS finder (Germany, Switzerland) (Figure 1, Table S1).

### 2.2 DNA extraction, GBS and SNP analysis

In this study, the modified cetyltrimethylammonium bromide (CTAB) method was used to extract DNA from plant needles (Tel‐zur et al., 1999). According to the method reported by Poland, Brown, et al. (2012) and Poland, Endelman, et al. (2012), the Genedenovo Bioinformatics Technology Co., Ltd. in Guangzhou, China, was responsible for the GBS. Each sample had 1.5 g of DNA extracted and digested using the restriction enzymes *EcoRI* and *NiaIII*. T4 DNA ligase (NEB) was used to add adapters to both ends of the digested DNA fragments, and the DNA was amplified. Electrophoresis was used to isolate and purify DNA fragments ranging in size from 400 to 600 base pairs. On the Illumina HiSeq 4000 sequencing platform (Illumina, CA, USA), 150 bp paired‐end reads were sequenced from the purified product.

To call single nucleotide polymorphisms (SNPs), we utilised the fastp program (Chen et al., 2018) to filter the raw data. In order to get clean data, we trimmed the reads, got rid of the 5′‐end linker sequences and got rid of the aberrant nucleotide bases and low‐quality ends of the reads (reads with ≥10% unidentified nucleotides and reads with >50% bases having Phred quality scores of ≤10). The ‘mem‐t4‐k32‐M’ command in Burrows‐Wheeler Aligner (BWA) v0.7.8‐r455 program (Li & Durbin, 2009) was implemented to map the clean data to the European spruce (*P. abies*) reference genome. After alignment, the Bayesian method in GATK software (McKenna et al., 2010) was applied for SNP calling of the population, and then the VariantFiltration component in GATK was used to filter the SNPs based on the percentage standard to obtain the SNP dataset. Finally, VCFtools v.0.1.11 (Danecek et al., 2011) was utilised for further filtering, and just high‐quality SNPs (minor allele frequency ≥ 0.02, max‐missing ≤0.5) were retained for phylogenetic and population analyses.

### 2.3 Phylogenetic tree and population genetics analysis

Maximum‐likelihood (ML) trees were constructed from high‐quality SNPs using the GTR + I + G model in IQTree v.1.6.10 software (Nguyen et al., 2015) with the number of bootstrap replicates set to 1000 with 1000 bootstrap repetitions to reconstruct genetic connections of the *P. asperata* complex at the genome‐wide level. The obtained tree files were uploaded into Interactive Tree of Life (ITOL) (https://itol.embl.de/itol.cgi) for editing and beautification.

The sample populations' structures were analysed using Admixture v.1.3.0 software (Alexander et al., 2009) with *K* values ranging from 2 to 10. Linkage disequilibrium among high‐quality SNPs was filtered using PLINK v.1.90. We utilised the genetic component coefficient (*Q*) for each sample across all populations to generate a population genetic structure matrix and then tested for convergence using cross‐validation to identify the best number of clusters. The population genetic structure matrices were visualised using the Pophelper package v. 2.3.1 in R v. 4.3.1. Principal component analysis (PCA) in GCTA v.1.93.3 software (Yang et al., 2011) was used to determine the genetic structure of the spruce population. The nucleotide diversity (*π*) of each population, Tajima's *D* and population genetic differentiation (*F*_ST_) between populations were calculated in the PopGenome package v.2.7.5 in R v.4.1.2. The *F*_ST_ scan was conducted with 100‐kb sliding windows and 10‐kb steps. The PCA and *F*_ST_ results were visualised using the ggplot2 package v. 3.4.3 in R v. 4.3.1.

### 2.4 Estimation of species divergence times

Divergence times of the *P. asperata* complex were estimated based on high‐quality SNPs utilising MCMCtree in PAML v.4.9 (Yang, 2007) with the ‘independent rates’ option and ‘GTR’ model. After a burn‐in of 1000 iterations, a Markov chain Monte Carlo simulation was run for 100,000 generations. Two secondary calibration points were used from an earlier investigation of *Picea*: divergence times of *P. pungens* and the eight spruce species (13.92–23.52 Mya) and divergence time at the root node of *P. wilsonii* and *P. neoveitchii* (10.03–16.89 Mya) (Shao et al., 2019). The output documents were analysed with Tracer v.1.7.1 (Rambaut et al., 2018) to check for convergence and make sure the effective sample size was more than 200.

### 2.5 Gene flow

To detect gene flow among the populations of *P. asperata* complex, we used TreeMix analysis and the ABBA‐BABA. The software PLINK v.1.90 (Purcell et al., 2007) (indep‐pairwise50 10 0.1) was employed to reduce linkage disequilibrium effects. The TreeMix v.1.13 programme (Pickrell & Pritchard, 2012) was used for the analysis, with the ‘‐global’ and ‘‐se’ options used to determine the mean standard error (SE) of the migration percentage and the ‘‐noss’ parameter used to avoid overcorrection for species with small sample numbers. The migration parameter (m) values were set ranging from 1 to 10, and each value was repeated five times. The R package OptM (https://cran.r‐project.org/web/packages/OPTM) was used to determine the best value for ‘m’ in the number of edges to migrate. Finally, the R script ‘plotting_funcs.R’, which comes with TreeMix v1.13, was used for plotting after the optimal ‘m’ value was determined. Dsuite v.0.5‐r44 was employed to run the ABBA‐BABA test (Malinsky et al., 2021). *P. pungens* and *P. jezoensis* were designated outgroups (O), *P. wilsonii* and *P. neoveitchii* were designated ancestors (P1), *P. retroflexa*, *P. asperata* and *P. crassifolia* were designated sister population I (P2) and *P. koraiensis*, *P. meyeri* and *P. mongolica* were designated sister population II (P3). Jackknife was employed to get the *Z* score for the ABBA‐BABA test; when the Z score > 3, the result was considered significant.

### 2.6 Environmental association analysis

#### 2.6.1 Selection of environmental variables

Distribution data for the eight spruce species in this study were obtained from the Global Biodiversity Information Facility (GBIF, https://www.gbif.org/), Chinese Virtual Herbarium (CVH, http://www.cvh.ac.cn/) and published literature. A total of 398 distribution records were obtained after SDMtools filtering (Table S2). The 19 selected contemporary bioclimatic factors were obtained from Worldeclim (www.worldclim.org) with a spatial resolution of 2.5 min. Data of 19 bioclimatic factors from 398 distribution points were extracted using the extract multi values to Points function in Arcgis v.10.7, and R version 4.3.1 was used to calculate correlations and remove one of the factors with |*r*| > .7. Multicollinearity was also assessed by calculating variance inflation factors (VIFs) with the Vegan package v.2.6.4 of R v.4.3.1 to ensure that the selected environmental factors had VIF values <10. Finally, based on Pearson's correlation coefficients and VIF, four bioclimatic factors were selected for the analyses (BIO3: isothermality; BIO5: max temperature of warmest month; BIO6: min temperature of coldest month; and BIO12: annual precipitation).

#### 2.6.2 Redundancy analysis (RDA)

RDA was performed using the Vegan package v.2.6.4 for R v.4.3.1 to assess the effect of environmental factors on genetic variation (SNPs), and then potential candidate SNPs were identified based on *p*‐value (*p* < .05). Candidate SNPs were mapped to the reference genome *P. abies* to identify candidate genes, and genes were subjected to Gene Ontology (GO) analysis and Kyoto Encyclopedia of Genes and Genomes (KEGG) analysis. Genes associated with environmental adaptation were aligned to the *Arabidopsis thaliana* proteome using Blastx, with an *E*‐value threshold set at 1e^−5^.

### 2.7 Selective sweep analyses

To discover the regional adaptive genetic variation of *P. asperata* complex, we compared populations of different species according to the phylogenetic tree with *P. wilsonii* and *P. neoveitchii* as background populations: (A) *P. koraiensis* versus the background populations; (B) *P. retroflexa* versus the background populations; (C) *P. asperata* and *P. crassifolia* versus the background populations; (D) *P. meyeri* versus the background populations; and (E) *P. mongolica* versus the background populations. To detect regions with significant selective sweeps, regions with high *π* radio (top 5%) and high *F*_ST_ (top 5%) were identified as regions with selection signals, where *π* and *F*_ST_ were calculated using VCFtools v0.1.11. After the genes in each selected region were determined, Gene Ontology (GO) enrichment analysis and Kyoto Encyclopedia of Genes and Genomes (KEGG) pathway enrichment analysis were performed, which enhanced the comprehension of the genes' biological functions.

## 3 RESULTS

### 3.1 GBS sequences

The GBS library of 200 *Picea* samples produced a total of 1059.17 Gb of raw data, and an overall of 1014.86 Gb of clean data was acquired by filtering low‐quality sequences, with an average data volume of 5.07 Gb per sample. The average GC content was 39.74%, the distribution was normal, Q20 ≥ 97.90% and Q30 ≥ 93.69%, indicating that the base quality of the sequences was high. The average alignment rate between the genomes of the 10 *Picea* species and the reference genome of *P. abies* was 97.07%, indicating that these 10 *Picea* species are highly similar to *P. abies*. GATK software was used to obtain a total of 15,716,973 unfiltered SNPs from the clean data. After screening out missing sites (<0.5) and according to minor allele frequencies (>0.02), there were still 1,678,480 high‐quality SNPs for use in follow‐up analysis.

### 3.2 Phylogenetic relationships and population structure analyses

**FIGURE 2:**
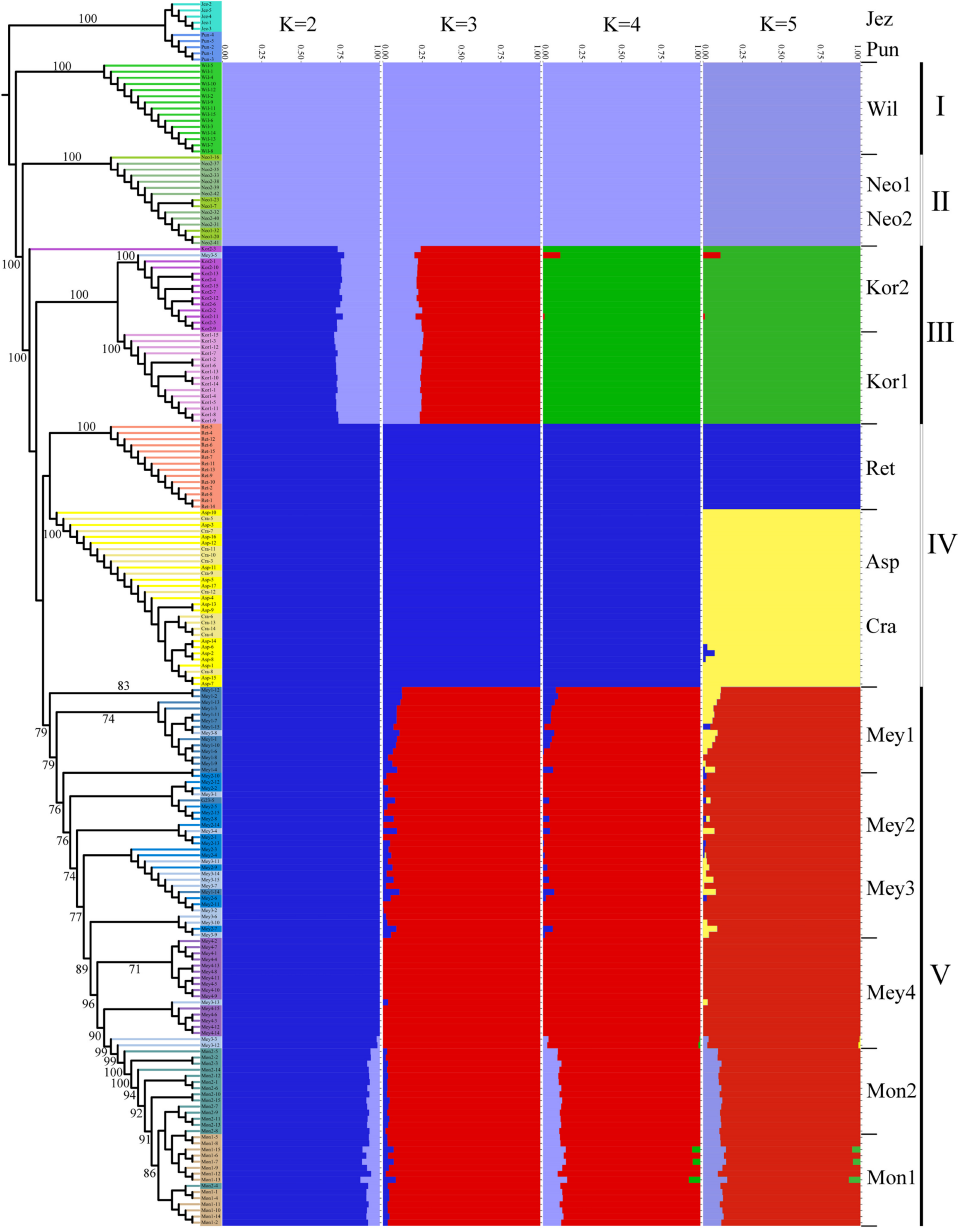
Maximum‐likelihood phylogenetic tree and STRUCTURE analysis of *Picea asperata* complex based on SNP markers. Pun: *P. pungens*; the remaining species' abbreviations are the same as in Figure 1.

**FIGURE 3:**
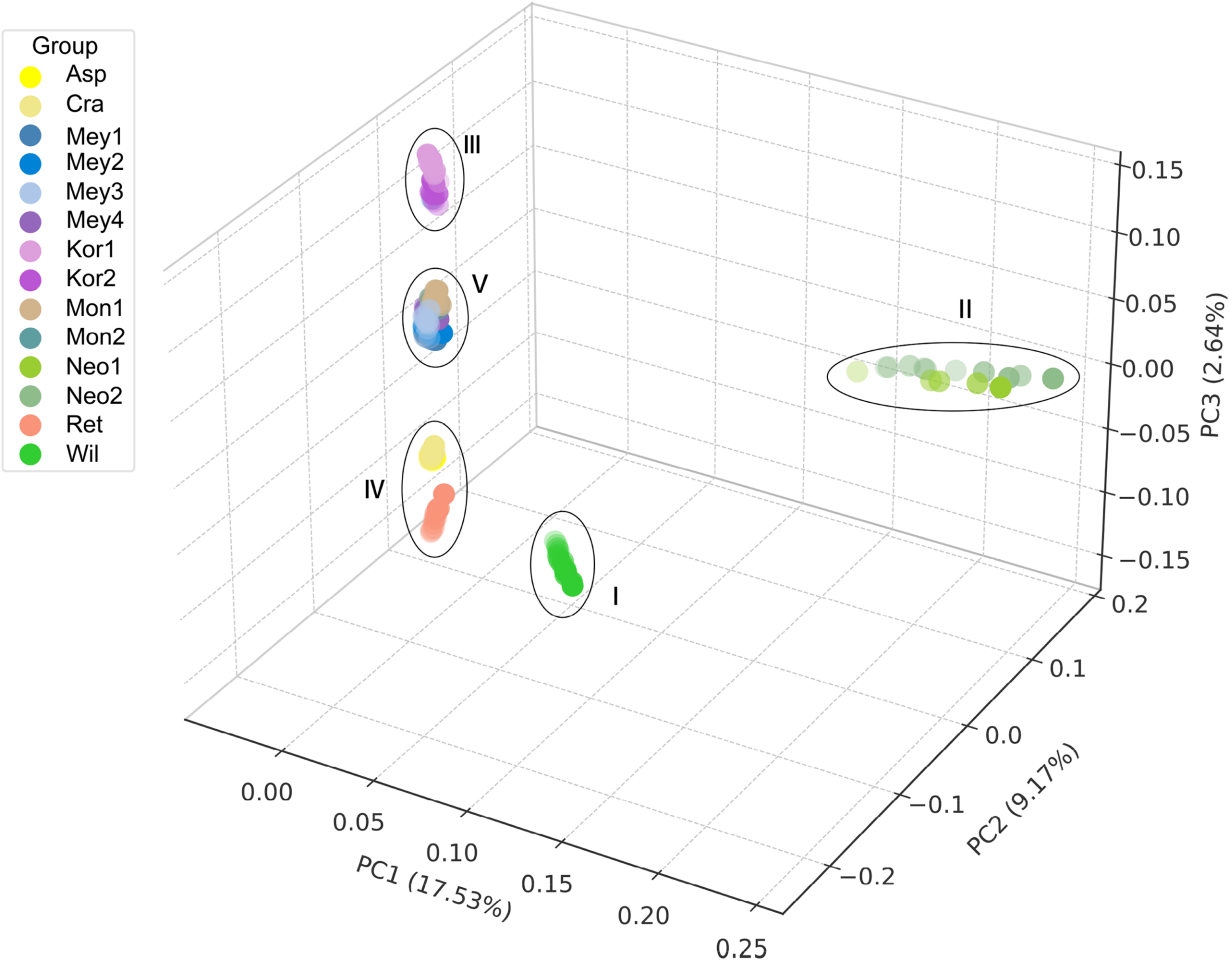
PCA analysis of *Picea asperata* complex. The species abbreviations are the same as in Figures 1 and 2.

As shown by the maximum‐likelihood tree in Figure 2, the 14 spruce populations can be divided into 5 populations (I–V) with *P. jezoensis* and *P. pungens* as outgroups, among which population I is the *P. wilsonii* population; population II includes the two populations of *P. neoveitchii*; population III consists of the two populations of *P. koraiensis*; population IV consists of populations of *P. retroflexa*, *P. crassifolia* and *P. asperata*; and population V consists of four populations of *P. meyeri* and two groups of *P. mongolica*. A subclade of population IV is composed of *P. crassifolia* and *P. asperata*, representing the tight relationship between the two species. In population V, the subclades of *P. meyeri* and *P. mongolica* can be well distinguished, and the population of *P. meyeri* sampled from Xiaowutai Mountain in Hebei Province is geographically close to the two populations of *P. meyeri* sampled in Shanxi and *P. meyeri* sampled on Wuling Mountain in Hebei Province. Therefore, the *P. meyeri* population from Xiaowutai Mountain was mixed with the *P. meyeri* population from Shanxi and the *P. meyeri* population from Wuling Mountain in the phylogenetic tree. We also further evaluated population structure under various *K* values (Figure 2). The CV error is smaller when *K* = 2–5, and reaches its minimum at *K* = 3 (Figure S1). When *K* = 2, *P. wilsonii* and *P. neoveitchii* are first clustered into an independent genetic component, while *P. koraiensis* contains genetic components from both *P. wilsonii* and *P. neoveitchii*. When *K* = 3, *P. retroflexa*, *P. asperata* and *P. crassifolia* differentiated into independent genetic components. Meanwhile, *P. koraiensis* contained the genetic components of both *P. wilsonii* and *P. neoveitchii*, as well as those of *P. meyeri* and *P. mongolica*. When *K* = 4, *P. koraiensis* differentiates to form an independent genetic component. When *K* = 5, *P. retroflexa* separates from *P. asperata* and *P. crassifolia*, forming an independent genetic component. When *K* ranges from 2 to 5, *P. mongolica* contains the genetic components of *P. wilsonii* and *P. neoveitchii*. When *K* ranges from 3 to 5, *P. meyeri* contains the genetic components of *P. retroflexa*, *P. asperata* and *P. crassifolia*. To further assess population structure, we utilised PCA to determine the relationships within spruce populations. As shown in Figure 3, the PCA clustering results are consistent with the phylogenetic tree results.

### 3.3 Molecular dating

**FIGURE 4:**
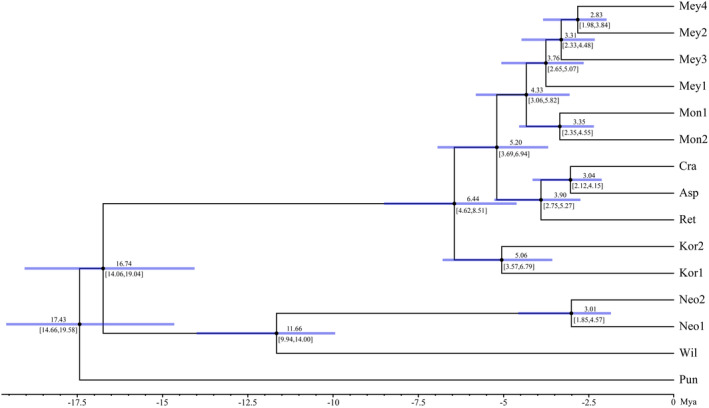
Estimation of divergence times of nine species of *Picea asperata* complex. The species abbreviations are the same as in Figures 1 and 2.

Figure 4 displays the outcomes of the SNP‐based divergence time estimation. The ancestor of *P. wilsonii* and *P. neoveitchii* was estimated to have diverged ~16.74 (95% confidence interval, 14.06–19.04) Mya, and the two species diverged ~11.66 (9.94–14.00) Mya. The *P. koraiensis* diverged at ~6.44 (4.62–8.51) Mya, followed by the ancestor of *P. meyeri* and *P. mongolica* diverged from the ancestor of *P. retroflexa*, *P. asperata* and *P. crassifolia* at ~5.20 (3.69–6.94) Mya. Then, the *P. meyeri* and *P. mongolica* diverged at ~4.33 (3.06–5.82) Mya.

### 3.4 Population gene flow analysis

**FIGURE 5:**
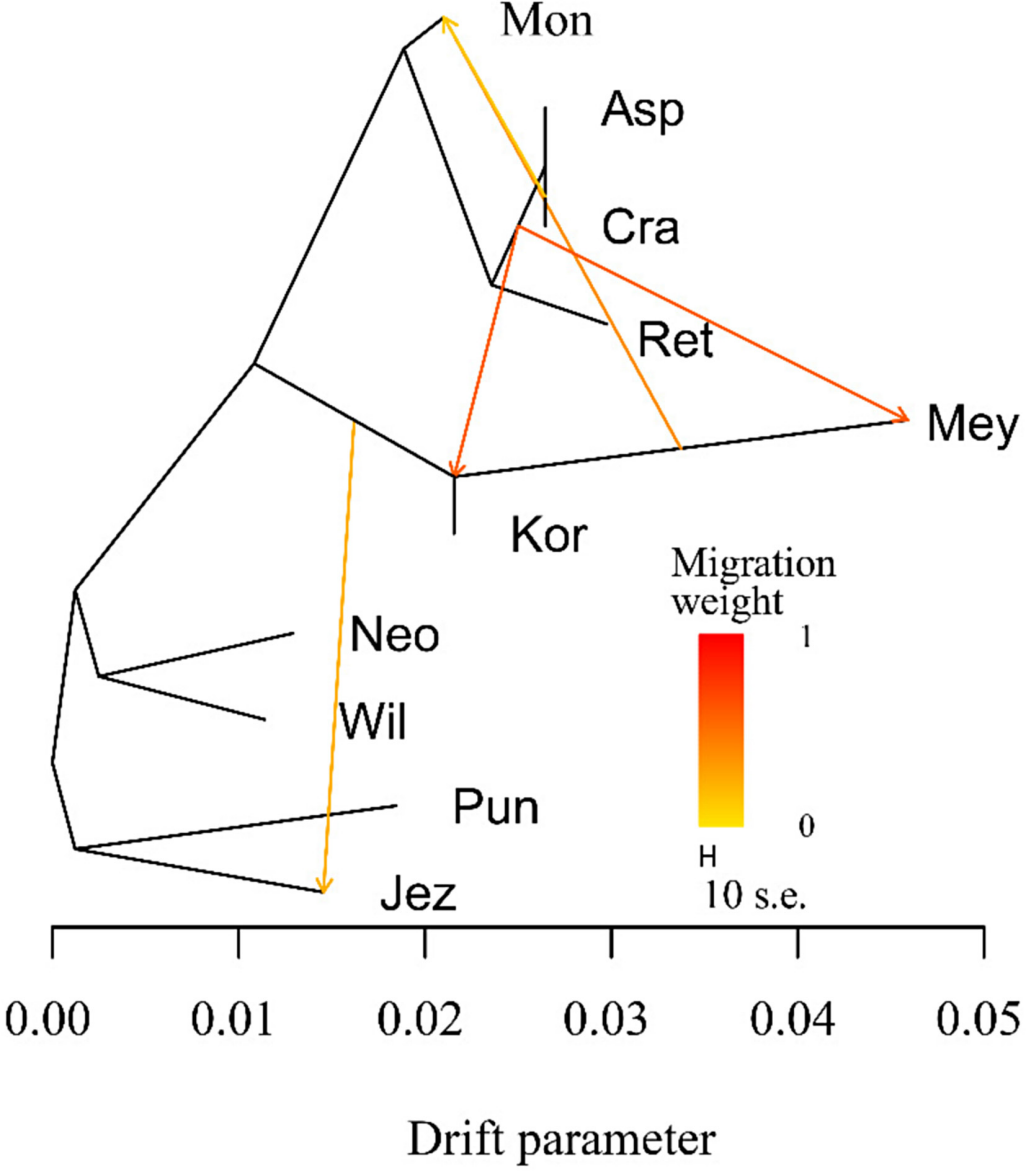
Interspecific gene flow of *Picea asperata* complex. Neo, *P. neoveitchii* from Gansu and Shaanxi; Kor, *P. koraiensis* from Gaofeng and Hailing; Mey, *P. meyeri* from Pangquangou, Mount Wutai, Xiaowutai Mountain and Wuling Mountain. Mon, *P. mongolica* from Baiyinaobao and Huamugou; the others are the same as in Figures 1 and 2.

**FIGURE 6:**
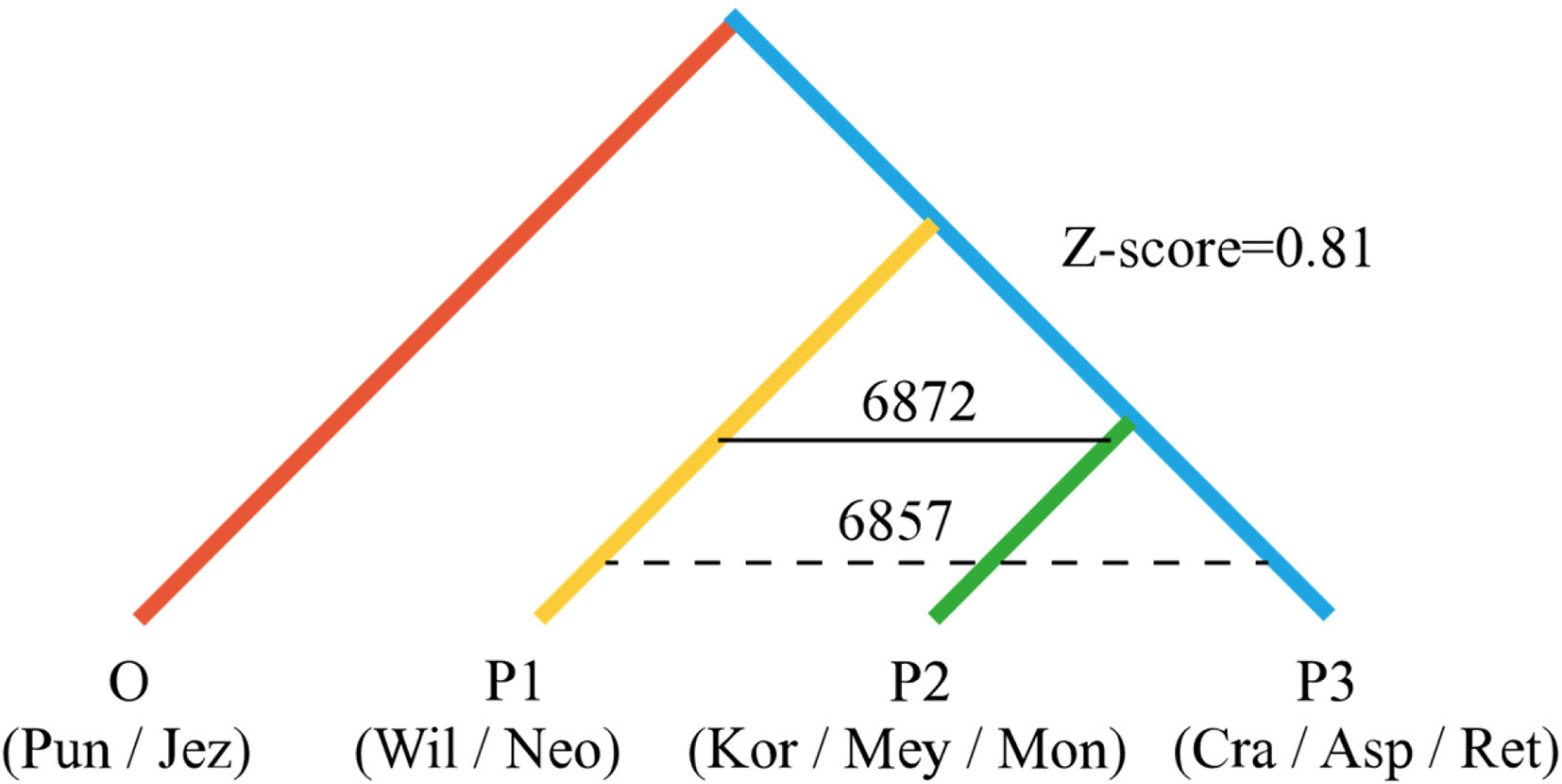
ABBA‐BABA test of *Picea asperata* complex. The species abbreviations are same as in Figure 5.

Maximum‐likelihood tree construction was performed in TreeMix and residual matrix of the *P. asperata* complex (Figure 5) to solve the problem of gene flow among populations. Migration events were added sequentially to the maximum‐likelihood tree. Then, the optimal number of migration edges was determined when m = 5 by the OptM package (Figure S2), indicating that there were five gene flow occurrences among species. There was one high‐weight instance of gene flow from the ancestor of *P. asperata* and *P. crassifolia* to the ancestor of *P. koraiensis* and *P. meyeri*. There was one instance of medium‐weight gene flow from the ancestor of *P. meyeri* to *P. mongolica*. There was also an instance of low‐weight gene flow from the ancestor of *P. crassifolia* to *P. mongolica* and from the ancestor of *P. koraiensis* and *P. meyeri* to *P. jezoensis*. This supports the result of admixture of genetic components between species in the structure analysis. To further confirm gene flow of *P. asperata* complex, we used the ABBA‐BABA test. Figure 6 shows that 6872 alleles were shared between P1 and P2, and 6857 alleles were shared between P1 and P3, with Z score = 0.81 < 3, indicating that there was no significant gene flow between P1 and the two sister populations P2 and P3. This further verified the results of the TreeMix analysis that there was no significant gene flow among *P. wilsonii*, *P. neoveitchii* and the other six spruce species.

### 3.5 Population genetic diversity and population genetic differentiation

To approximate the genetic diversity of the *P. asperata* complex, we computed the nucleotide diversity (*π*) at the genome‐wide level (Table 1). Within the *P. asperata* complex, *P. mongolica* exhibits the highest nucleotide diversity (*π* value), followed by *P. meyeri*, while *P. neoveitchii* has the lowest, with *P. wilsonii* slightly higher. Tajima's *D* can be used to assess whether nucleotide diversity deviates significantly from neutrality. The mean values of Tajima's *D* for the *P. asperata* complex were all greater than 0 (Table 1). Among these, *P. neoveitchii* had the highest mean Tajima's *D* value of 1.008422, while *P. crassifolia* had the lowest at 0.414048. The *π* value of *P. neoveitchii* was the lowest, while its Tajima's *D* was the highest, indicating that it may have recently experienced a population bottleneck.

**TABLE 1:** Estimation of genetic variation in eight spruce species.

Population	Nucleotide diversity (*π*)	Tajima's *D*
Wil	2.37 × 10^−4^	0.556024
Neo	2.24 × 10^−4^	1.008422
Kor	4.02 × 10^−4^	0.684063
Ret	3.19 × 10^−4^	0.694321
Asp	3.74 × 10^−4^	0.506791
Cra	3.56 × 10^−4^	0.414048
Mey	4.08 × 10^−4^	0.694879
Mon	4.22 × 10^−4^	0.532124

The species abbreviations are the same as in Figure 2.

**FIGURE 7:**
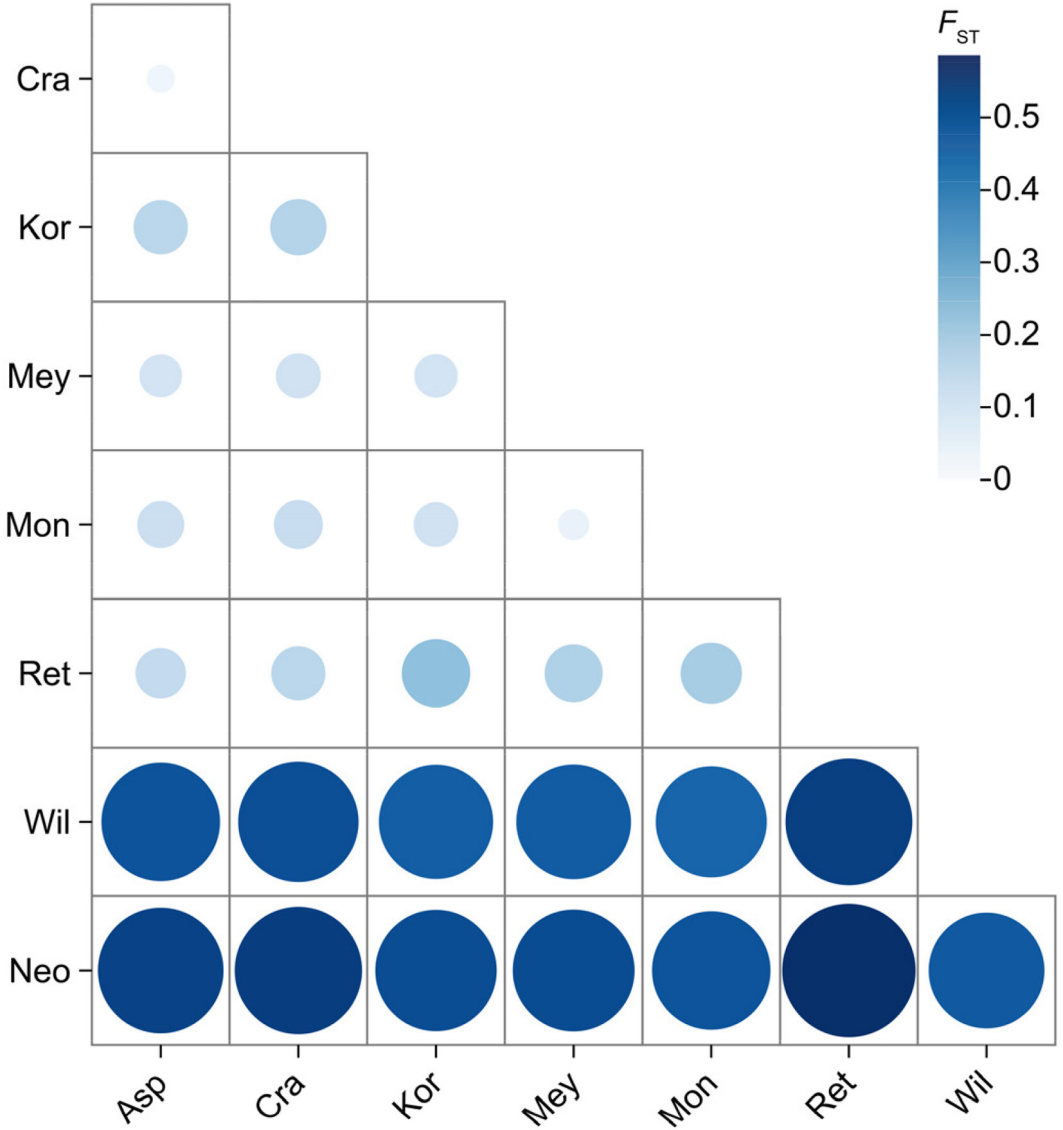
Interspecific genetic differentiation among eight spruce species. The species abbreviations are the same as in Figure 2.

According to the results of population genetic differentiation (*F*_ST_) analysis among the *P. asperata* complex shown in Figure 7, the degree of interspecific differentiation of *P. neoveitchii* and *P. wilsonii* was relatively high, and the interspecific differentiation of the other six spruce species was relatively low. This indicates that *P. koraiensis*, *P. meyeri*, *P. mongolica*, *P. asperata*, *P. crassifolia* and *P. retroflexa* are closely related. Particularly, *P. meyeri* and *P. mongolica*, as well as *P. asperata* and *P. crassifolia*, show especially close relationships.

### 3.6 Genetic variation of environmental adaptability in spruce

**FIGURE 8:**
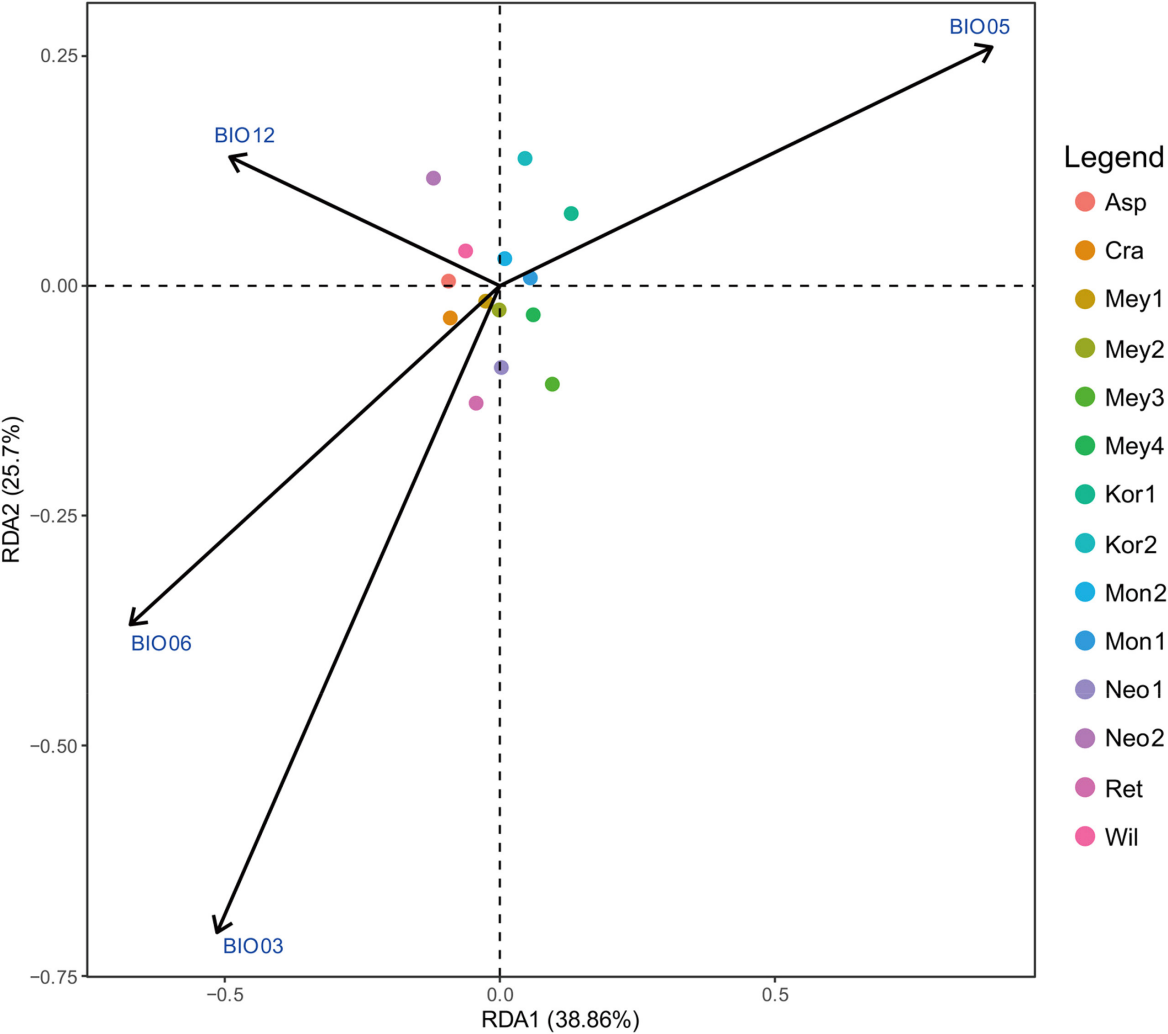
RDA analysis of genetic variation in eight spruce species and environmental variables. The species abbreviations are the same as in Figure 2.

As shown in Figure 8, Figure S3, a total of 120,178 SNPs were confirmed to be related to environmental variables BIO3, BIO5, BIO6 and BIO12. These SNPs may be variations produced by different spruce species to adapt to the local environment. The first three RDA axes explained 38.86%, 25.70% and 19.69% of the total variation respectively. The genetic variation of *P. Neoveitchii* (Shaanxi), *P. wilsonii* and *P. asperata* was most affected by annual precipitation (BIO12). Isothermality (BIO03) and min temperature of coldest month (BIO06) explained most of the genetic variation in *P. crassifolia*, *P. meyeri*, *P. Neoveitchii* (Gansu) and *P. retroflexa*. Max temperature of warm month (BIO5) contributed the most to the genetic variation of *P. koraiensis* and *P. mongolica*.

We used RDA analysis to identify a total of 20,808 potential candidate genes associated with environmental variables in eight spruce species (Table S3). From Go enrichment analysis (Figure S4), it can be seen that the molecular functions of potential candidate genes were mainly related to protein binding, GTPase binding, binding and protein dimerisation activity. KEGG analyses showed that the candidate gene pathways were mainly focused on endocytosis, flavonoid biosynthesis and ether lipid metabolism (Figure S5). In addition, comparing these candidate genes with the *Arabidopsis* proteome through BLASTX, many proteins related to environmental adaptation were found, such as disease resistance protein (TAO1, RPP1, etc.); receptor‐like protein kinase family proteins (FLS2, RCH2, etc.), which may be involved in the response of plants to adversity (Narusaka et al., 2009; Zou et al., 2018); cytochrome P450 superfamily protein (TT7), which is involved in UV photoprotection, facilitates species acclimatisation to high‐altitude environments (Ryan et al., 2001); terpenoid cyclases/protein prenyltransferases superfamily protein (GA1), which is related to the flower morphology (Brock et al., 2012); and HAESA‐like 1 (HSL1) is associated with seed longevity and leaf epidermal cell development (Chen et al., 2022; Roman et al., 2022).

### 3.7 Selective sweep analysis of spruce

Selective sweep analysis reveals the adaptation characteristics of population evolution by comparing the selected genes between populations. In this study, *P. neoveitchii* and *P. wilsonii* were used as the background populations, and five subgroups were compared with the background populations. A total of 537 genes that underwent selection during species differentiation were identified through the combined strategy of the top 5% each of *π* and *F*_ST_ values. Among them, the selected areas of *P. koraiensis* showed *F*_ST_ ≥ 0.84 and *π*_Wil/Neo_/*π*_Kor_ ≥ 4.16, the selected areas of *P. retroflexa* showed *F*_ST_ ≥ 0.86 and *π*_Wil/Neo_/*π*_Ret_ ≥ 6.28, the selected areas of *P. asperata* and *P. crassifolia* showed *F*_ST_ ≥ 0.85 and *π*_Wil/Neo_/*π*_Asp/Cra_ ≥ 5.94, the selected areas of *P. meyeri* showed *F*_ST_ ≥ 0.84 and *π*_Wil/Neo_/*π*_Mey_ ≥ 4.49 and the selected areas of *P. mongolica* showed *F*_ST_ ≥ 0.83 and *π*_Wil/Neo_/*π*_Mon_ ≥ 3.59 (Figure S6).

**FIGURE 9:**
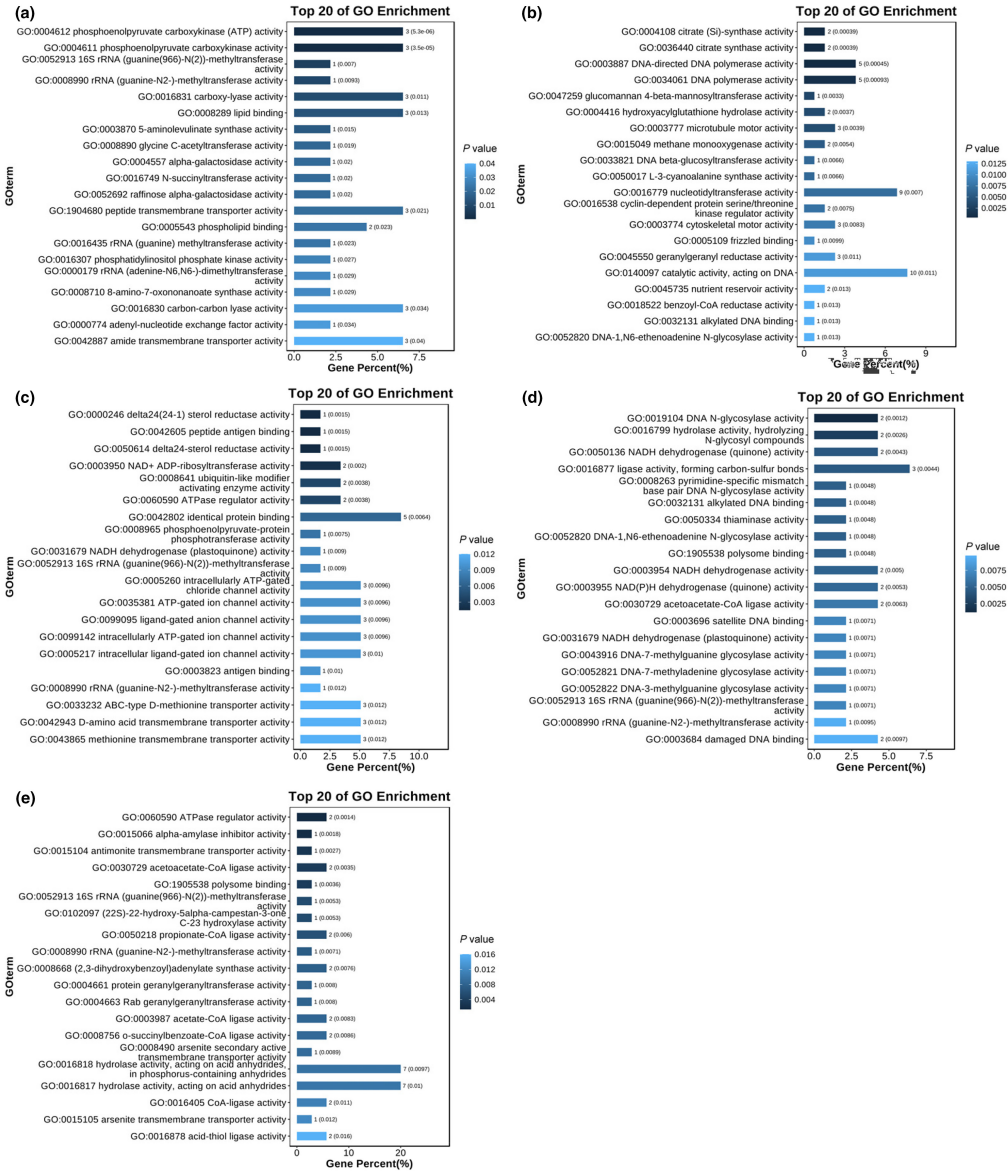
GO enrichment analysis of genes experiencing selective sweeps. (a) Genes in *Picea koraiensis*; (b) Genes in *P. retroflexa*; (c) Genes in *P. asperata* and *P. crassifolia*; (d) Genes in *P. meyeri*; (e) Genes in *P. mongolica*.

**FIGURE 10:**
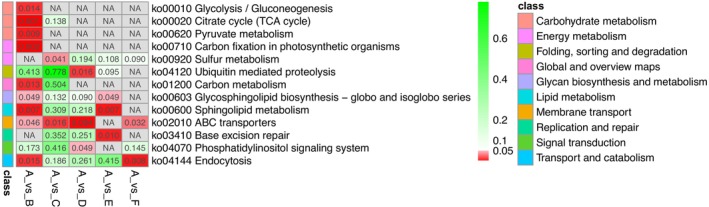
Heatmap for KEGG analysis of selected genes in spruce. A: *Picea wilsonii* and *P. neoveitchii*; B: *P. koraiensis*; C: *P. retroflexa*; D: *P. asperata*/*P. crassifolia*; E: *P. meyeri*; F: *P. mongolica*.

*Picea koraiensis* had 73 selected genes (Table S4). Based on the GO analysis (Table S5), these selected genes were annotated with 108 terms, of which 66 belonged to biological processes (BP), 19 to cellular components (CC) and 23 to molecular functions (MF). The molecular functions are primarily enriched in activities such as phosphoenolpyruvate carboxykinase (ATP) activity, lipid binding and amide transmembrane transporter activity, among others (Figure 9a); KEGG enrichment analysis (Figure 10) revealed that the majority of the genes were enriched in citrate cycle (TCA cycle) and carbon fixation in photosynthetic organisms.

*Picea retroflexa* has 231 selected genes (Table S6). Based on the GO enrichment analysis (Table S7), these genes were annotated with 345 terms, allocated as follows: 234 in BP, 44 in CC and 67 in MF. The molecular functions are primarily enriched in activities such as catalytic activity, acting on DNA, DNA polymerase activity and citrate synthase activity, among others (Figure 9b). KEGG analysis (Figure 10) showed that the genes were mainly enriched in ABC transporters and sulphur metabolism.

*Picea asperata* and *P. crassifolia* had 101 selected genes (Table S8). Based on the GO analysis (Table S9), these genes were annotated with 392 terms, with 276 belonging to BP, 22 to CC and 94 to MF. The molecular functions are primarily enriched in NADH dehydrogenase activity, damaged DNA binding and alkylated DNA binding, among others (Figure 9c). KEGG analysis (Figure 10) showed that the genes were mainly enriched in ABC transporters and ubiquitin‐mediated proteolysis.

*Picea meyeri* had 78 selected genes (Table S10). Based on the GO enrichment analysis (Table S11), these genes were annotated with 224 terms, with 150 terms in BP, 18 in CC and 56 in MF. The molecular functions were predominantly enriched in ubiquitin‐like modifier‐activating enzyme activity, methionine transmembrane transporter activity, peptide antigen binding, etc. (Figure 9d). KEGG analysis (Figure 10) revealed that the majority of genes were enriched in sphingolipid metabolism and base excision repair.

*Picea mongolica* had 54 selected genes (Table S12). Based on the GO enrichment analysis (Table S13), these genes were annotated with 244 terms, with distributions of 175 in BP, 22 in CC and 47 in MF. The molecular functions are primarily enriched in ATPase regulator activity, alpha‐amylase inhibitor activity and antimonite transmembrane transporter activity, among others (Figure 9e). The genes were primarily enriched in endocytosis and ABC transporters, according to KEGG analysis (Figure 10).

A gene (*HK5*) related to plant growth and development, a gene (*HMGB7*) related to heat stress, a gene (*LNK1*) related to circadian rhythm and two genes (*SC35*, *RIC1*) related to plant flowering were found among the selected genes of each subgroup. *P. retroflexa* and *P. crassifolia/P. asperata* showed the greatest duplication of selected genes, with a total of 72 genes, which are mainly related to plant stress resistance (*MED15A*, *HMGB7*, *TFT7*, *UBA2*, *LIP5*, *GLOX*, *HSR201*, *HUB2*) and associated with plant chloroplasts (*GGPS1*, *ISE2*, *MRL7*, *DOT4*). There are 32 identical selected genes in *P. meyeri* and *P. mongolica*, primarily related to plant stress tolerance (*SKD1*, *MSH6*, *HMGB7*, *HSP70‐17*). These results indicated that due to the geographical environmental differences in distribution areas among different *Picea* species, the selected target genes were all involved in flowering and climate adaptation. *P. retroflexa* and *P. crassifolia/P. asperata* are similar in geographical distribution and the similarity of target genes selected during differentiation is higher than in other comparisons.

## 4 DISCUSSION

### 4.1 Phylogenetic and gene flow features of *P. asperata* complex

The effective population size of *Picea* is large, and the generation time is long, which makes the pedigree screening process slow. At the same time, studies have shown that *Picea* is derived from recent radiation‐based differentiation (Leslie et al., 2012). These characteristics make the phylogenetic relationships more complex. Zheng and Fu (1978) divided the spruce distributed in China into Sect. *Picea*, Sect. *Casicta* and Sect. *Omorica* according to the shape of leaves and the number of stomatal lines. Plant phylogenetic background, as well as abiotic and biotic environments, are potential factors of phenotypic variation in plants (Ackerly, 2004; Watanabe et al., 2007). Interspecific differences in traits such as those of needles, seeds and cones in *Picea* are driven by climate factors, and these traits exhibit a wide range of variation along a water gradient (Li et al., 2016). Consequently, it is challenging to accurately reflect the relationships between spruce species based solely on phenotypic characteristics. The phylogenetic tree constructed from the whole genome and PCA analysis in this study revealed that *P. wilsonii* and *P. neoveitchii* are distantly related to other species within *P. asperata* complex. Additionally, in the STRUCTURE analysis, *P. wilsonii* and *P. neoveitchii* initially formed independent genetic clusters. This finding aligns with the results of phylogenetic trees based on transcriptome data previously reported by Feng et al. (2019) and Shao et al. (2019). In the ML tree constructed in this study, *P. asperata* and *P. crassifolia* were closely related, and they were clustered into a subclade with *P. retroflexa*. In the STRUCTURE analysis, *P. asperata* and *P. crassifolia* consistently clustered into one genetic cluster, while *P. retroflexa* was not separated into an independent genetic cluster until *K* = 5. Whereas in the phylogenetic tree generated by Feng et al. (2019) from transcriptome data, *P. retroflexa* and *P. asperata* were sister species, and *P. crassifoli*a and *P. koraiensis* were sister species. The results of Shao et al. (2019) are similar to ours, with *P. crassifolia* and *P. retroflexa* being sister species to each other and clustered as a subclade with *P. asperata*. The high levels of gene flow between different species of *Picea* and their reticulate evolution, relatively close distribution ranges of *P. asperata*, *P. crassifolia* and *P. retroflexa*, and sampling of a small number of samples in fewer regions will cause bias in genetic relationship analysis. In the phylogenetic tree, *P. meyeri* and *P. mongolica* are sister species and are clustered into a single genetic cluster in the STRUCTURE analysis, indicating a close relationship between the two. The genetic clusters of *P. meyeri* collected from Shanxi and Xiaowutai Mountain in Hebei are mixed with those of *P. asperata*, *P. crassifolia* and *P. retroflexa*. In contrast, the clusters from Wuling Mountain in Hebei are less mixed, reflecting the geographical distribution. Genetic clusters of *P. wilsonii* and *P. neoveitchii* are found within *P. mongolica*. However, gene flow analysis reveals no significant gene flow among the three species, a phenomenon that may be attributed to incomplete lineage sorting within *Picea*.

*Picea koraiensis*, *P. retroflexa*, *P. crassifolia*, *P. asperata*, *P. meyeri* and *P. mongolica* are closely related and have similar phenotypic traits. Moreover, the *π* value of these six spruce species is higher than that of *P. wilsonii* and *P. neoveitchii*, and the *F*_ST_ among species is smaller, which may be because many genetic variations are shared throughout the species, and there is no sign of complete reproductive isolation (Feng et al., 2019). Gene flow among species frequently leads to phenotypic convergence (Ran et al., 2015). Our TreeMix analysis also showed four instances of gene flow among those six spruce species, and there was also an instance of low‐weight gene flow from the ancestor of *P. koraiensis* and *P. meyeri* to *P. jezoensis*, which may have been responsible for gene flow among ancestral species when *Picea* spread from North America to Asia across the Bering Land Bridge.

Various geological events in geological history have promoted the evolution of species on large spatial scales (Crisci, 2001). The molecular clock results indicate that *P. wilsonii* and *P. neoveitchii* diverged the earliest among the eight spruce species, which may be related to the rapid uplift of the western Qinling Mountains at ~16 Ma due to the northeastern growth of the Qinghai–Tibet Plateau (Wang et al., 2012), whereas the divergence of the two may have been affected by the uplift of Taibai Mountain at 8–15 Mya and the accelerated exhumation of Daba Mountain in the late Miocene (Heberer et al., 2014; Yang et al., 2017). The differentiation of *P. koraiensis* may be related to the uplift of Daxinganling, NE China, during the late Miocene (Li et al., 2021). The divergence time of the ancestors of *P. meyeri* and *P. mongolica* from the ancestors of *P. retroflexa, P. asperata* and *P. crassifolia* coincided with the stepwise uplift of the northeastern Qinghai–Tibetan Plateau during the late Miocene (Li et al., 2014). The differentiation time of *P. meyeri* and *P. mongolica* coincided with the rapid uplift of the Mongolian Plateau at 5 ± 3 Mya (Vassallo et al., 2007). These geographic isolations produced by mountain range formation and plateau uplift have facilitated rapid species differentiation in the *P. asperata* complex.

### 4.2 Genomic differentiation and signatures of selective sweeps

The fossil record and some recent genomic studies suggest that *Picea* originated in western North America and spread to northern Asia across the Bering Land Bridge at least twice during the Miocene and Pliocene. Then, due to lower temperatures, some species migrated southwards to refuges and rapidly expanded their distribution areas during the interglacial and postglacial periods (Ran et al., 2015). Due to the different ecological environments in different regions, mutations in species constantly allow adaptation to different site conditions, which accelerates species divergence. Whereas temperature and precipitation are key determinants of species distribution and growth (Root et al., 2003; Wahid et al., 2007), redundancy analyses showed that isothermality, max temperature of warmest month, min temperature of coldest month and annual precipitation contributed most of the genetic variation associated with environmental adaptation in the *P. asperata* complex. *P. neoveitchii*, *P. wilsonii and P. asperata* tend to prefer wet environments, and annual precipitation has a significant impact on them; *P. meyeri*, *P. crassifolia*, *P. neoveitchii* and *P. retroflexa* are not resistant to low temperatures, and temperature fluctuations and low temperatures affect their distribution areas; the distribution areas of *P. koraiensis* and *P. mongolica* are cold and dry in winter, so high‐temperature and humid areas limit their distribution. It is precisely because of the differences in the adaptability of spruce to the environment that the phenomenon of species substitution of *Picea* in different regions has occurred. In this study, we also found that the genes selected in *P. asperata* complex under environmental stress are primarily associated with maturation, development and reaction to stress. Despite the fact that selective sweeps have a tendency to reduce the genetic diversity of a particular population, they will enhance the adaptability of the species to the environment. At the same time, for entire species, as alleles have varying distribution ranges, selective sweeps have the potential to fix them in various locations, which will result in an increase in genomic diversity overall. (Coop et al., 2009). In this study, *P. wilsonii* and *P. neoveitchii* were used as background populations, and the genes that underwent selective sweeps in *P. koraiensis*, *P. retroflexa*, *P. crassifolia*, *P. asperata*, *P. meyeri* and *P. mongolica* were mainly associated with plant stress tolerance, growth and development. The *HK5* gene has important effects on root growth, leaf width, inflorescence architecture and flower development (Burr et al., 2020). The *LNK1* gene is responsible for photoperiod‐dependent flowering, circadian rhythms and photomorphogenic responses in plants (Rugnone et al., 2013). The *RIC1* gene does not only control pollen tube development (Zhou et al., 2015) but also act as a positive regulator of the auxin response and a negative regulator of the ABA response to control seedling root development (Choi et al., 2013). The *SC35* gene affects the splicing of *FLC* intron 1 and transcription of *FLC* to regulate flowering in *Arabidopsis* (Yan et al., 2017), and expression of the *HMGB7* gene is upregulated when *Agrostis scabra* is subjected to heat stress (Xu & Huang, 2018). Despite the small number of *Picea* species, the genus is broadly distributed in the Northern Hemisphere. There are some differences in the phenology of *Picea* in different distribution areas; thus, among the selected genes, there were genes related to the regulation of plant rhythms, photoperiod response and flowering. At the same time, we also found a gene related to heat stress (*HMGB7*) among the selected genes in six spruce species compared with the background group. This is probably because spruce is mostly distributed at middle and high latitudes or in alpine and subalpine regions, and temperature is one of the key climatic elements that plays a vital role in determining its dispersion (Li et al., 2022; Liu, Cao, et al., 2022). Compared with the background populations, *P. crassifolia*, *P. asperata* and *P. retroflexa* have a higher upper altitude limit, and *P. koraiensis* is distributed at higher latitudes, while *P. mongolica* is distributed mainly on the more arid eastern edge of the Otingdag sandy land. The distribution altitude of *P. meyeri* is higher than that of *P. neoveitchii*, which is similar to that of *P. wilsonii*, but *P. wilsonii* can be distributed in lower‐latitude areas. Therefore, these six spruce species are weaker in high‐temperature tolerance than the two spruce species constituting the background population, *P. wilsonii* and *P. neoveitchii*. The genes under selective sweeps in *P. wilsonii* and *P. likiangensis* as defined by Liu, Qin, et al. (2022) and the genes in *Juglans regia* and *J. sigillata* reported by Ji et al. (2021) involved in blooming and withstanding stress were found. Furthermore, in addition to the *HMGB7* gene, we also identified the plant heat tolerance‐related gene *HIT4* in *P. koraiensis*, which is mainly distributed in northeastern China at higher latitudes compared to the background populations, and the temperature difference between the two distribution regions is relatively large. Genes related to plant epidermal cells (*ATML1* and *HTH*), plant trichomes (*RR21* and *CPL3*) and lignin regulation (*IRL*) were found in *P. retroflexa*. *P. retroflexa* is mostly distributed in alpine areas at altitudes of 3000–3800 m (Fu et al., 1999), where ultraviolet radiation is also stronger, and the epidermis of leaves and bark are the interface between plants and the external environment, which helps plants resist ultraviolet radiation and avoid DNA damage. Genes related to plant leaf colour (*MYB6* and *ERF017*) and leaf morphology development (*SLC1* and *DTX54*) were also identified in *P. retroflexa*, and leaf morphology and colour are important morphological features for the classification of spruce species. In *P. crassifolia/P. asperata*, genes related to vascular bundle development (*ATHB‐15*) and DNA damage repair (*PARP2*) were found, which may be related to the altitude distribution. *P. crassifolia* and *P. asperata* are mainly distributed in high‐altitude areas, where ultraviolet rays can cause greater damage to plant DNA, and vascular bundles help plants resist ultraviolet rays. A gene associated with root growth (*APSR1*) and a gene associated with plant height (*CYP90D2*) were found in *P. meyeri* and *P. mongolica* respectively. *APSR1* can regulate the normal growth of plant roots under phosphate starvation stress (Víctor et al., 2013). It was also found that *P. mongolica* and *P. wilsonii*/*P. neoveitchii* differed significantly from one another in terms of the height of their plants. Selective sweeps drive species divergence within genera by rapidly immobilising favourable mutant genes, as well as enhancing the adaptation of species to new environments, and this rapid adaptation is generally achieved by selective removal of multiple loci (Coop et al., 2009).

## 5 CONCLUSIONS

The 14 populations of *P. asperata* complex are divided into 5 branches, with *P. crassifolia* and *P. asperata*, as well as *P. meyeri* and *P. mongolica*, being closely related and each pair clustering into one genetic cluster respectively. *P. wilsonii* and *P. neoveitchii* diverged earlier, exhibit lower gene diversity, are more distantly related to the rest of *P. asperata* complex and show no significant gene flow. The uplift of the Qinling Mountains facilitated the differentiation of *P. wilsonii* and *P. neoveitchii* from *P. asperata* complex. Similarly, the Miocene uplift of the Daxinganling led to the differentiation of *P. koraiensis*. Additionally, the differentiation of *P. meyeri*, *P. mongolica*, *P. retroflexa*, *P. asperata* and *P. crassifolia* was influenced by the uplift of both the Qinghai–Tibet Plateau and the Mongolian Plateau. Although the uplift of the mountains has created geographic isolation, frequent gene flow still occurs among *P. koraiensis*, *P. meyeri*, *P. mongolica*, *P. asperata*, *P. crassifolia* and *P. retroflexa*. Local adaptability has fostered interspecies differentiation within *P. asperata* complex, with 20,808 potential candidate genes related to environmental adaptation being identified. Selective sweep analysis revealed that the genes under selection pressure are primarily associated with plant stress resistance and organ development, which enhances the adaptability of spruce to environmental changes and accelerates interspecies differentiation. The research findings enhance our understanding of species differentiation and environmental adaptability within the *Picea* genus. Future studies could verify the functions of selected genes using the genetic transformation system of *Picea*.

## AUTHOR CONTRIBUTIONS

**Yifu Liu:** Conceptualization (equal); formal analysis (equal); investigation (equal); resources (equal); software (equal); writing – original draft (lead); writing – review and editing (equal). **Wenfa Xiao:** Investigation (equal); resources (equal); writing – review and editing (equal). **Fude Wang:** Funding acquisition (equal); resources (equal). **Ya Wang:** Formal analysis (equal); investigation (equal); resources (equal); software (equal). **Yao Dong:** Investigation (equal); software (equal). **Wen Nie:** Software (equal). **Cancan Tan:** Software (equal). **Sanping An:** Funding acquisition (supporting); resources (equal). **Ermei Chang:** Writing – review and editing (equal). **Zeping Jiang:** Investigation (equal). **Junhui Wang:** Conceptualization (equal); data curation (equal); supervision (equal); writing – review and editing (equal). **Zirui Jia:** Conceptualization (lead); data curation (equal); funding acquisition (lead); project administration (lead); supervision (equal); writing – review and editing (lead).

## FUNDING INFORMATION

The work was financially supported by the National Key Research and Development Program of China (2023YFD2200605‐02) and the National Natural Science Foundation of China (31500540).

## CONFLICT OF INTEREST STATEMENT

The authors declare no conflicts of interest.

## CONSENT TO PARTICIPATE

This research was conducted in compliance with relevant Chinese laws.

## Supporting information


Figure S1

Table S1

## Data Availability

The raw sequencing data generated from this study have been deposited in NCBI SRA (https://www.ncbi.nlm.nih.gov/sra) under the accession number PRJNA876367.
