# Supplementary material for: Species divergence and environmental adaptation of Picea asperata complex at the whole genome level
Source: Ecol Evol. 2024 Aug 6;14(8):e70126. doi: 10.1002/ece3.70126 (PMC11303459; doi:10.1002/ece3.70126)
Supplement: Supplementary file 1 — Figure S1 [file ECE3-14-e70126-s002.doc]

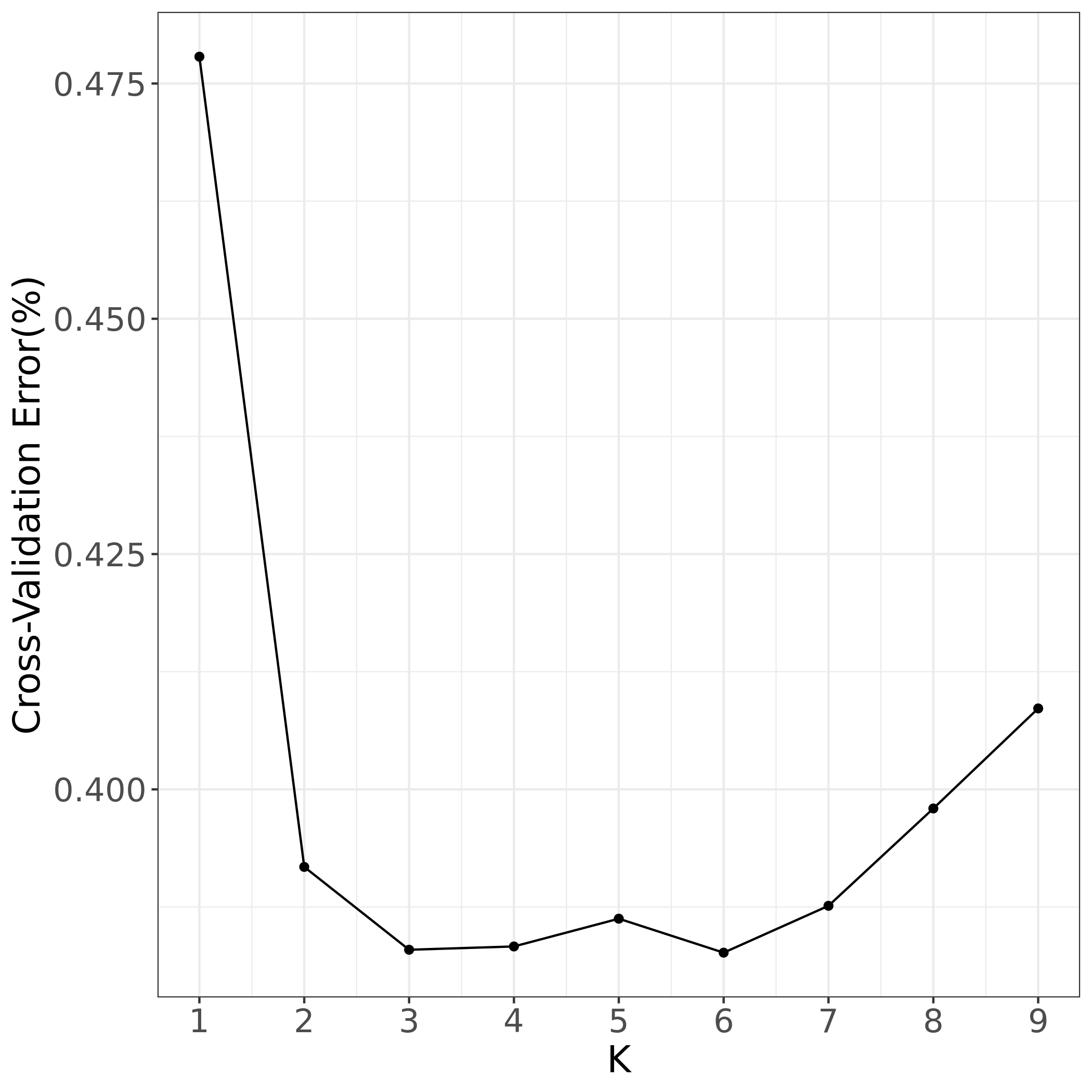


Figure S1 Cross-validation error value


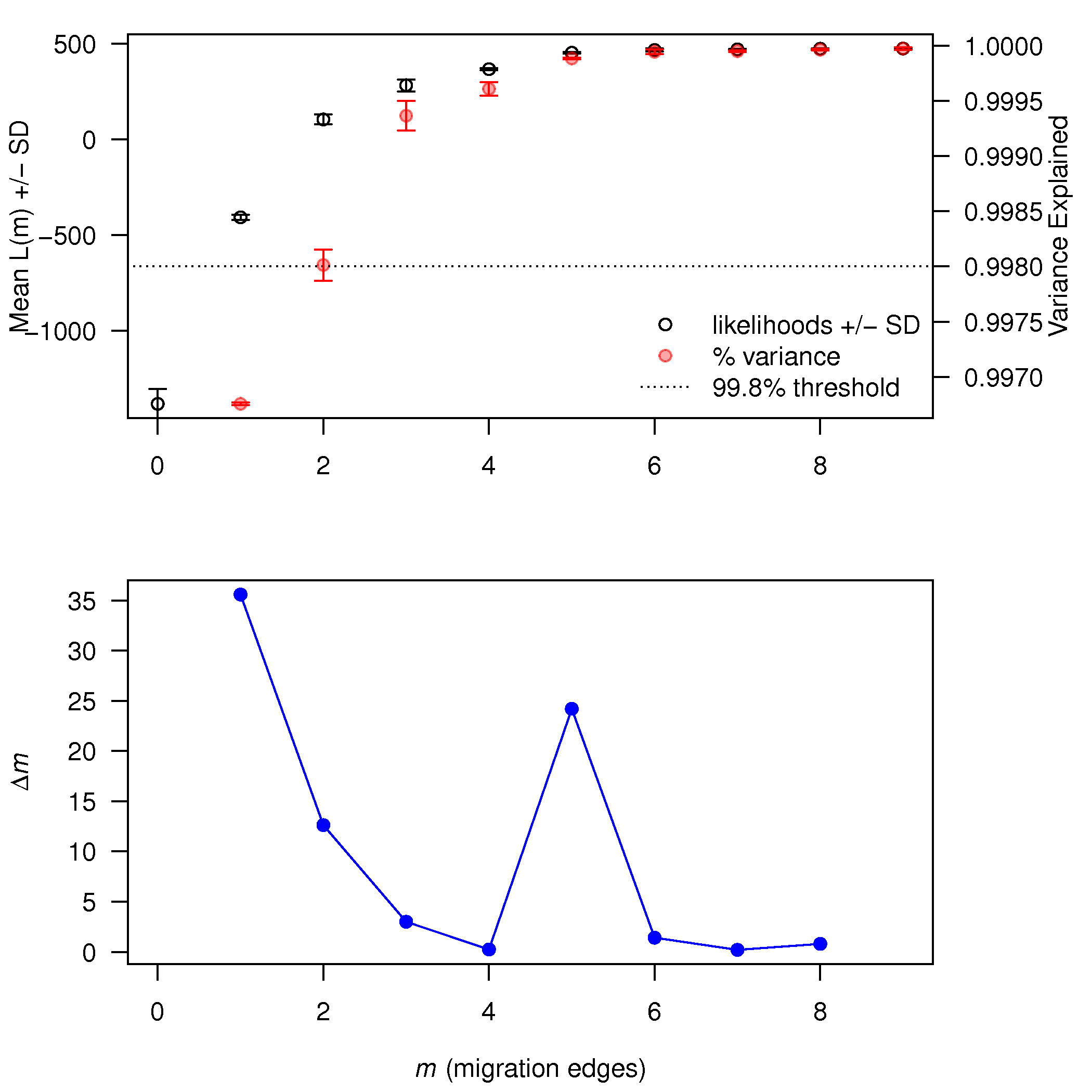


Figure S2 Optimal number of migration edges


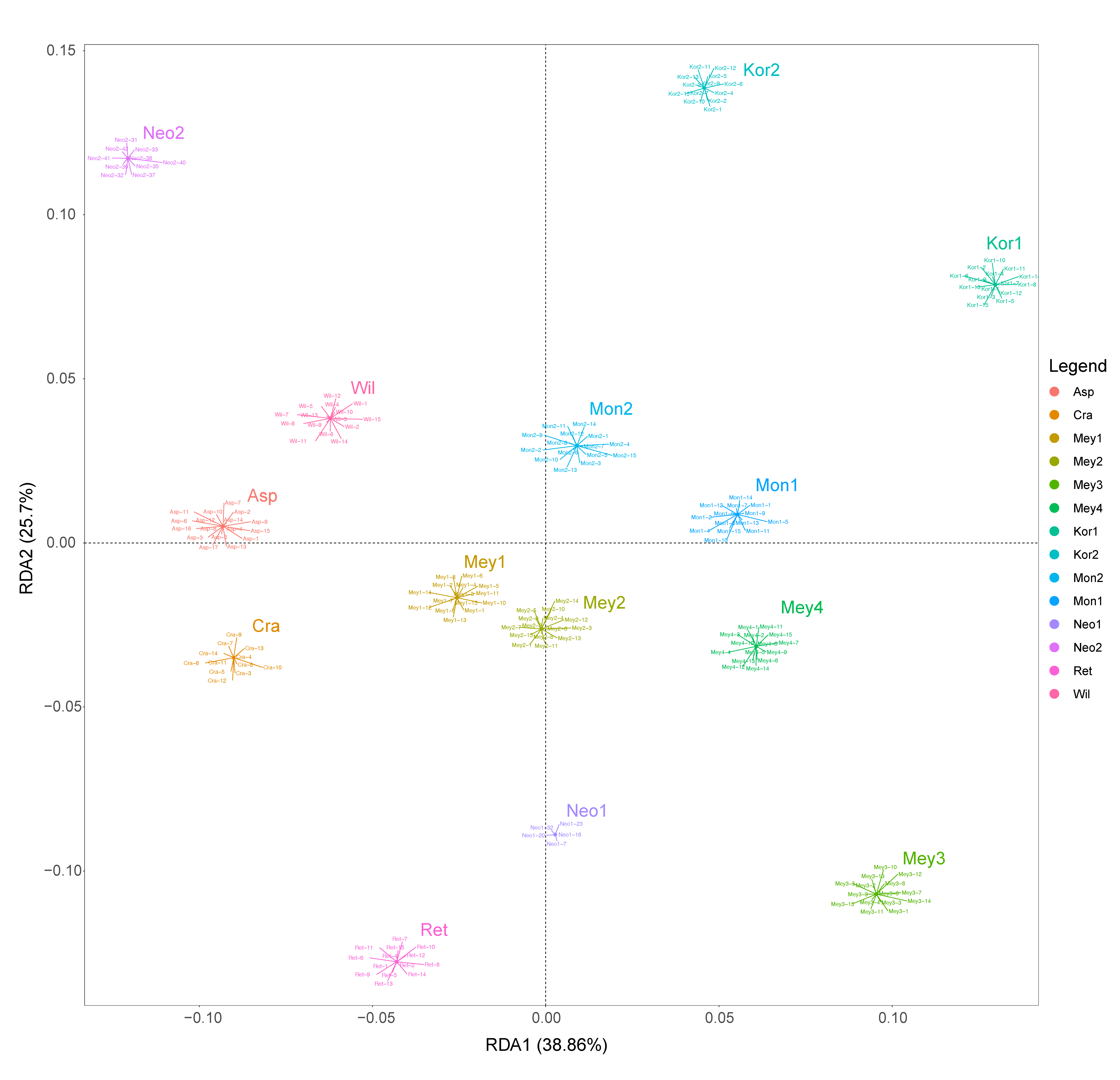


Figure S3 RDA analysis of genetic variation in eight spruce species and environmental variables. Jez: *P. jezoensis*; Pun: *P. pungens*; Wil: *P. wilsonii*; Neo1, Neo2: *P. neoveitchii* from Gansu and Shaanxi, respectively; Kor1, Kor2: *P. koraiensis* from Gaofeng and Hailing, respectively; Ret: *P. retroflexa*; Cra: *P. crassifolia*; Asp: *P. asperata*; Mey1, Mey2, Mey3, Mey4: *P. meyeri* from Pangquangou, Mount Wutai, Xiaowutai Mountain and Wuling Mountain, respectively; Mon1, Mon2: *P. mongolica* from Baiyinaobao and Huamugou, respectively.


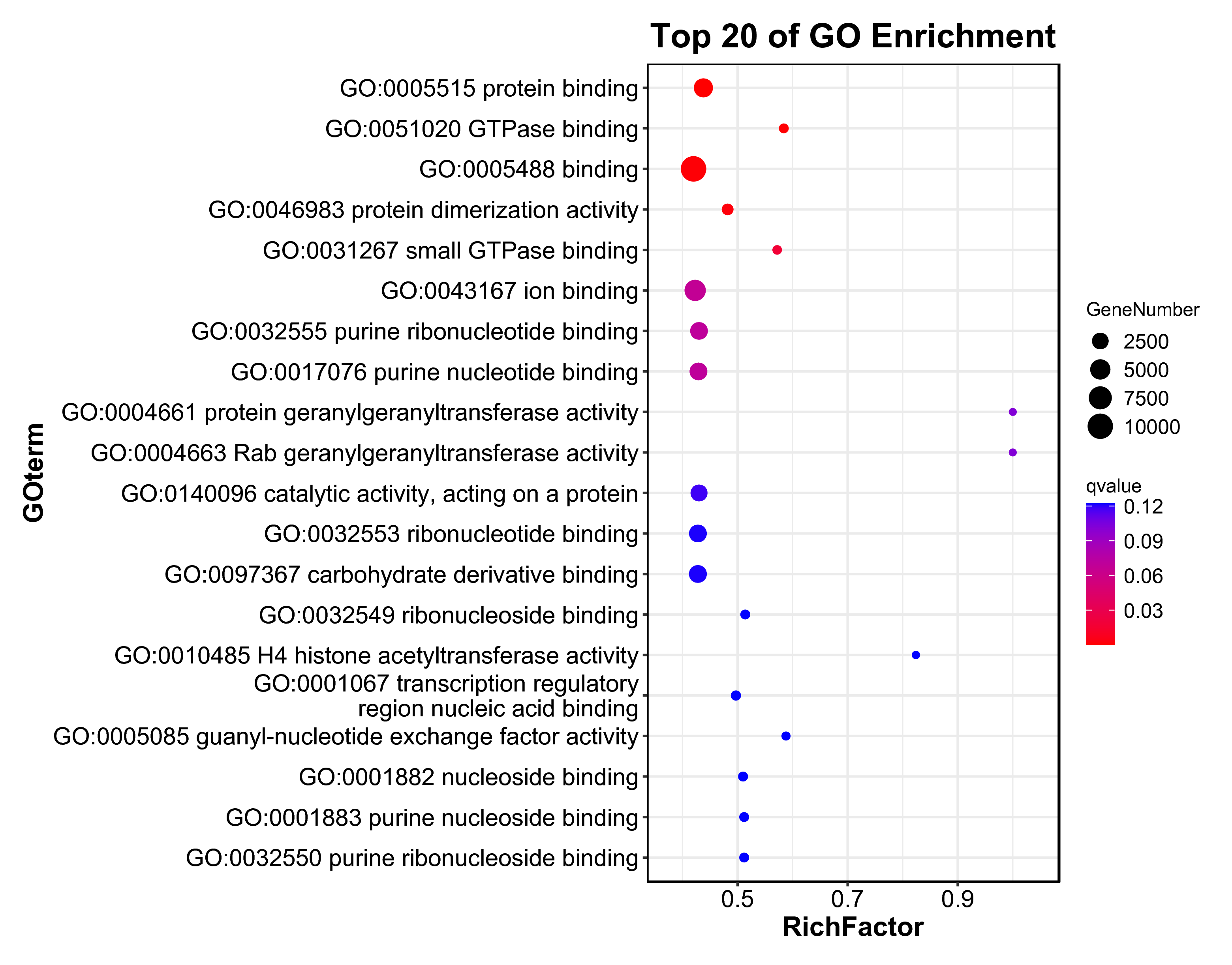


Figure S4 Map for GO enrichment analysis of potential candidate genes related to environmental variables in spruce.


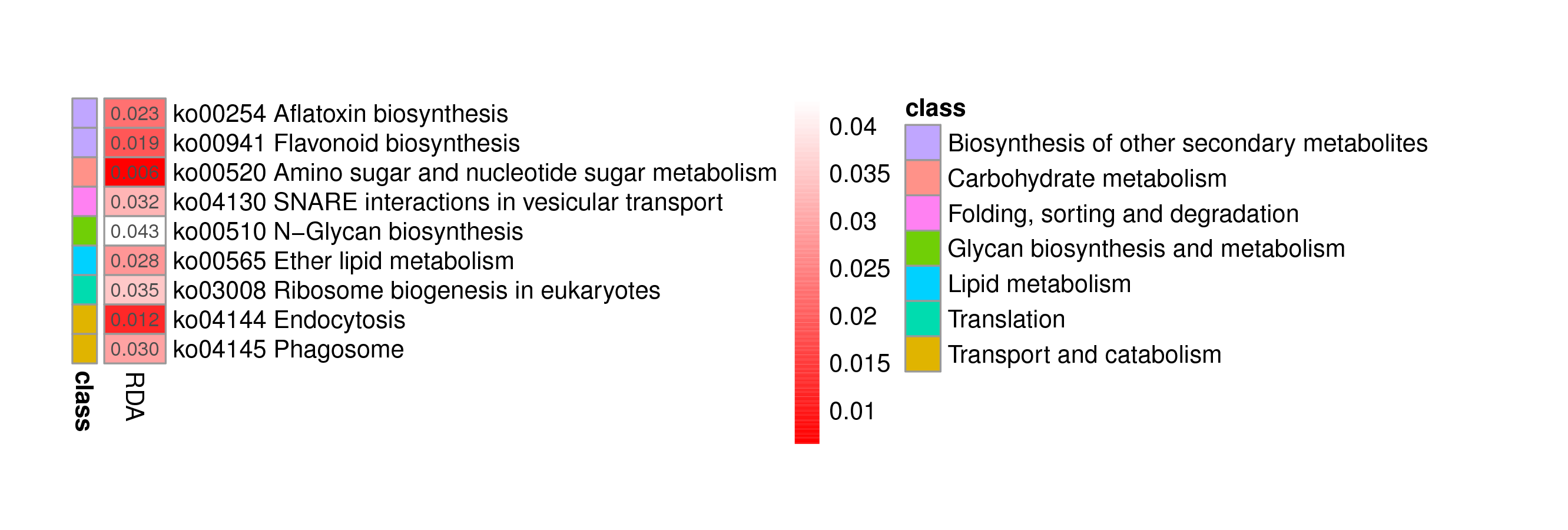


Figure S5 Heatmap for KEGG analysis of potential candidate genes related to environmental variables in spruce.





Figure S6 Genomic regions with selective sweep in *Picea*. A, *P wilsonii* / *P. neoveitchii* versus *P. koraiensis*; B, *P wilsonii* / *P. neoveitchii* versus *P. retroflexa*; C, *P wilsonii* / *P. neoveitchii* versus *P. asperata* / *P. crassifolia*; D, *P wilsonii* / *P. neoveitchii* versus *P. meyeri*; E, *P wilsonii* / *P. neoveitchii* versus *P. mongolica*.
