# Supplementary material for: Species divergence and environmental adaptation of Picea asperata complex at the whole genome level
Source: Ecol Evol. 2024 Aug 6;14(8):e70126. doi: 10.1002/ece3.70126 (PMC11303459; doi:10.1002/ece3.70126)
Supplement: Supplementary file 2 — Table S1 [file ECE3-14-e70126-s001.zip › Table S1-S2S9.docx]

Table S1 Geographic information of the spruce populations in the study

| Population | Species | Sampling location | Longitude (E) | Latitude (N) |
| --- | --- | --- | --- | --- |
| Kor1 | *Picea koraiensis* | Gaofeng, Nenjiang, Heilongjiang, China | 125.270122E | 49.097204N |
| Kor2 | *Picea koraiensis* | Hailing, Heilongjiang, China | 128.319405E | 44.260865N |
| Mon1 | *Picea mongolica* | Baiyinaobao, Keshiketeng, Inner Mongolia, China | 117.213026E | 43.529202N |
| Mon2 | *Picea mongolica* | Huamugou, Keshiketeng, Inner Mongolia, China | 117.406612E | 42.651882N |
| Mey1 | *Picea meyeri* | Pangquangou, Jiaocheng, Shanxi, China | 111.556793E | 37.882106N |
| Mey2 | *Picea meyeri* | Wutai Mountain, Xinzhou, Shanxi, China | 113.595708E | 38.976198N |
| Mey3 | *Picea meyeri* | Xiaowutai Mountain, Yuxian, Hebei, China | 114.9396139E | 39.94055848N |
| Mey4 | *Picea meyeri* | Wuling Mountain, Xinglong, Hebei, China | 117.494444E | 40.595756N |
| Cra | *Picea crassifolia* | Datong, Qinghai, China | 101.837407E | 37.047215N |
| Asp | *Picea asperata* | Jiuzhaigou, Jiuzhaigou County, Sichuan, China | 103.905401E | 33.152679N |
| Wil | *Picea wilsonii* | Shenchi, Xinzhou, Shanxi, China | 112.088596E | 38.930839N |
| Ret | *Picea retroflexa* | Daofu, Sichuan, China | 101.32455E | 30.786099N |
| Neo1 | *Picea neoveitchii* | Bailong River, Zhouqu, Gansu, China | 104.106016E | 33.861973N |
| Neo2 | *Picea neoveitchii* | Taibai, Shaanxi, China | 107.74056E | 34.04017N |
| Jez | *Picea jezoensis* | Mengkeshan, Tahe, Heilongjiang, China | 124.309677E | 52.630183N |
| Pun | *Picea pungens* | New York, America | 74.3397W | 40.8982N |

Table S2 Distribution sites of eight *Picea* relatives

| *Species* | Latitude (N) | Longitude (E) | Species | Latitude (N) | Longitude (E) |
| --- | --- | --- | --- | --- | --- |
| *Picea asperata* | 101.2708 | 30.8125 | *Picea mongolica* | 116.6875 | 42.8542 |
| *Picea asperata* | 101.2708 | 31.7292 | *Picea mongolica* | 116.7708 | 43.6458 |
| *Picea asperata* | 101.5208 | 30.2708 | *Picea mongolica* | 116.8542 | 42.8958 |
| *Picea asperata* | 101.5625 | 32.6458 | *Picea mongolica* | 116.8542 | 43.6875 |
| *Picea asperata* | 102.3125 | 28.8542 | *Picea mongolica* | 116.8958 | 43.6875 |
| *Picea asperata* | 102.6875 | 31.8125 | *Picea mongolica* | 117.1458 | 43.3125 |
| *Picea asperata* | 102.8125 | 31.5625 | *Picea mongolica* | 117.1875 | 43.3542 |
| *Picea asperata* | 102.9375 | 31.5208 | *Picea mongolica* | 117.1875 | 43.5208 |
| *Picea asperata* | 102.9375 | 32.1042 | *Picea mongolica* | 117.1875 | 43.5625 |
| *Picea asperata* | 103.1458 | 33.6042 | *Picea mongolica* | 117.1875 | 43.6042 |
| *Picea asperata* | 103.3542 | 34.5625 | *Picea mongolica* | 117.2292 | 42.5625 |
| *Picea asperata* | 103.5208 | 32.3958 | *Picea mongolica* | 117.2292 | 42.8125 |
| *Picea asperata* | 103.5625 | 32.7708 | *Picea mongolica* | 117.2292 | 43.4792 |
| *Picea asperata* | 103.6458 | 32.8958 | *Picea mongolica* | 117.2292 | 43.5208 |
| *Picea asperata* | 103.6875 | 32.8958 | *Picea mongolica* | 117.2292 | 43.5625 |
| *Picea asperata* | 103.8958 | 32.7708 | *Picea mongolica* | 117.2292 | 43.8542 |
| *Picea asperata* | 105.8958 | 31.1875 | *Picea mongolica* | 117.2708 | 43.5625 |
| *Picea asperata* | 107.9375 | 33.3958 | *Picea mongolica* | 117.3125 | 43.8958 |
| *Picea asperata* | 108.5625 | 33.7708 | *Picea mongolica* | 117.3542 | 43.8125 |
| *Picea asperata* | 109.7292 | 30.1875 | *Picea mongolica* | 117.3542 | 43.8542 |
| *Picea crassifolia* | 100.2708 | 38.1458 | *Picea mongolica* | 117.3958 | 42.6458 |
| *Picea crassifolia* | 100.2708 | 38.2292 | *Picea mongolica* | 117.3958 | 43.4375 |
| *Picea crassifolia* | 100.3125 | 38.1875 | *Picea mongolica* | 117.4375 | 43.3125 |
| *Picea crassifolia* | 100.6042 | 34.6458 | *Picea mongolica* | 117.5208 | 43.6042 |
| *Picea crassifolia* | 100.8125 | 34.7292 | *Picea mongolica* | 117.6458 | 43.7708 |
| *Picea crassifolia* | 100.8125 | 34.7708 | *Picea neoveitchii* | 102.4792 | 34.6042 |
| *Picea crassifolia* | 101.0208 | 36.6458 | *Picea neoveitchii* | 104.3542 | 33.5625 |
| *Picea crassifolia* | 101.1458 | 36.7292 | *Picea neoveitchii* | 104.3958 | 33.5625 |
| *Picea crassifolia* | 101.6875 | 36.8958 | *Picea neoveitchii* | 106.8125 | 33.6875 |
| *Picea crassifolia* | 101.6875 | 37.0208 | *Picea neoveitchii* | 107.5208 | 33.8125 |
| *Picea crassifolia* | 101.8542 | 35.2292 | *Picea neoveitchii* | 107.7708 | 33.7708 |
| *Picea crassifolia* | 102.1458 | 37.1875 | *Picea neoveitchii* | 107.8958 | 33.8542 |
| *Picea crassifolia* | 102.8542 | 35.1042 | *Picea neoveitchii* | 108.4375 | 34.0208 |
| *Picea crassifolia* | 102.8542 | 35.1458 | *Picea neoveitchii* | 108.8125 | 32.0208 |
| *Picea crassifolia* | 103.1875 | 33.6042 | *Picea neoveitchii* | 109.7708 | 30.1875 |
| *Picea crassifolia* | 103.3542 | 34.5625 | *Picea neoveitchii* | 109.7708 | 30.2292 |
| *Picea crassifolia* | 103.5208 | 34.5625 | *Picea neoveitchii* | 109.8958 | 31.5208 |
| *Picea crassifolia* | 103.7292 | 34.1458 | *Picea neoveitchii* | 111.0625 | 31.5208 |
| *Picea crassifolia* | 103.7292 | 34.9375 | *Picea neoveitchii* | 111.3958 | 31.3542 |
| *Picea crassifolia* | 103.8125 | 34.0625 | *Picea neoveitchii* | 111.9375 | 33.4792 |
| *Picea crassifolia* | 104.4792 | 33.6042 | *Picea retroflexa* | 100.4375 | 34.9792 |
| *Picea crassifolia* | 105.8542 | 38.9792 | *Picea retroflexa* | 100.4792 | 33.1042 |
| *Picea crassifolia* | 105.8958 | 38.6875 | *Picea retroflexa* | 100.7292 | 31.3125 |
| *Picea crassifolia* | 105.8958 | 38.9792 | *Picea retroflexa* | 100.8125 | 31.8125 |
| *Picea crassifolia* | 106.2708 | 37.2708 | *Picea retroflexa* | 100.8125 | 34.7292 |
| *Picea crassifolia* | 107.4375 | 33.8542 | *Picea retroflexa* | 101.1875 | 31.8125 |
| *Picea crassifolia* | 111.1458 | 40.8958 | *Picea retroflexa* | 101.3125 | 30.7708 |
| *Picea crassifolia* | 98.2708 | 36.3542 | *Picea retroflexa* | 101.3542 | 34.6042 |
| *Picea crassifolia* | 98.5208 | 34.9375 | *Picea retroflexa* | 101.3958 | 32.5625 |
| *Picea crassifolia* | 99.8125 | 35.5208 | *Picea retroflexa* | 101.5208 | 29.8125 |
| *Picea crassifolia* | 99.8542 | 35.5208 | *Picea retroflexa* | 101.5625 | 29.4792 |
| *Picea koraiensis* | 121.3958 | 46.5208 | *Picea retroflexa* | 101.7292 | 29.5208 |
| *Picea koraiensis* | 121.6042 | 51.4375 | *Picea retroflexa* | 101.8125 | 30.8958 |
| *Picea koraiensis* | 123.1875 | 52.6042 | *Picea retroflexa* | 101.8542 | 27.8125 |
| *Picea koraiensis* | 123.3125 | 52.1875 | *Picea retroflexa* | 101.9375 | 35.3542 |
| *Picea koraiensis* | 123.3542 | 52.3542 | *Picea retroflexa* | 101.9792 | 29.9375 |
| *Picea koraiensis* | 124.2292 | 52.6458 | *Picea retroflexa* | 102.1458 | 31.4792 |
| *Picea koraiensis* | 124.4792 | 51.8125 | *Picea retroflexa* | 102.5208 | 29.7708 |
| *Picea koraiensis* | 124.5208 | 51.8125 | *Picea retroflexa* | 102.6458 | 30.3958 |
| *Picea koraiensis* | 124.5208 | 52.3125 | *Picea retroflexa* | 102.6458 | 32.0208 |
| *Picea koraiensis* | 124.6458 | 52.3542 | *Picea retroflexa* | 102.6875 | 31.8542 |
| *Picea koraiensis* | 125.2292 | 49.4375 | *Picea retroflexa* | 102.8958 | 31.1042 |
| *Picea koraiensis* | 125.4375 | 49.1458 | *Picea retroflexa* | 103.3958 | 33.4375 |
| *Picea koraiensis* | 126.1042 | 44.5208 | *Picea retroflexa* | 103.5208 | 31.5625 |
| *Picea koraiensis* | 126.1875 | 44.4792 | *Picea retroflexa* | 103.5625 | 31.8125 |
| *Picea koraiensis* | 126.8125 | 49.6042 | *Picea retroflexa* | 103.9375 | 33.1458 |
| *Picea koraiensis* | 127.2292 | 41.9792 | *Picea retroflexa* | 104.7292 | 32.5208 |
| *Picea koraiensis* | 127.2292 | 48.7708 | *Picea wilsonii* | 100.0625 | 30.8125 |
| *Picea koraiensis* | 127.3125 | 49.8125 | *Picea wilsonii* | 100.3125 | 32.2708 |
| *Picea koraiensis* | 127.5208 | 45.1875 | *Picea wilsonii* | 100.7292 | 31.5625 |
| *Picea koraiensis* | 127.6458 | 42.0625 | *Picea wilsonii* | 100.9792 | 32.2708 |
| *Picea koraiensis* | 128.0208 | 46.6875 | *Picea wilsonii* | 101.2708 | 27.9375 |
| *Picea koraiensis* | 128.0625 | 46.6875 | *Picea wilsonii* | 101.6042 | 36.4792 |
| *Picea koraiensis* | 128.0625 | 48.3125 | *Picea wilsonii* | 101.6875 | 32.1458 |
| *Picea koraiensis* | 128.1042 | 42.3125 | *Picea wilsonii* | 101.6875 | 36.9375 |
| *Picea koraiensis* | 128.1875 | 42.1875 | *Picea wilsonii* | 101.7292 | 36.9375 |
| *Picea koraiensis* | 128.1875 | 42.2292 | *Picea wilsonii* | 101.7708 | 35.8542 |
| *Picea koraiensis* | 128.4792 | 47.5208 | *Picea wilsonii* | 101.8542 | 35.9375 |
| *Picea koraiensis* | 128.5208 | 48.0208 | *Picea wilsonii* | 101.9375 | 29.9792 |
| *Picea koraiensis* | 128.5625 | 44.4375 | *Picea wilsonii* | 101.9375 | 36.3542 |
| *Picea koraiensis* | 128.5625 | 47.6042 | *Picea wilsonii* | 101.9792 | 30.0625 |
| *Picea koraiensis* | 128.6042 | 47.6042 | *Picea wilsonii* | 101.9792 | 36.3958 |
| *Picea koraiensis* | 128.6458 | 48.1042 | *Picea wilsonii* | 102.0208 | 36.8542 |
| *Picea koraiensis* | 128.7708 | 47.6042 | *Picea wilsonii* | 102.1458 | 36.8542 |
| *Picea koraiensis* | 128.7708 | 47.6458 | *Picea wilsonii* | 102.3125 | 31.7292 |
| *Picea koraiensis* | 128.8542 | 47.1875 | *Picea wilsonii* | 102.4375 | 36.9375 |
| *Picea koraiensis* | 128.8958 | 47.1875 | *Picea wilsonii* | 102.4792 | 35.6875 |
| *Picea koraiensis* | 128.8958 | 47.6875 | *Picea wilsonii* | 102.4792 | 36.9375 |
| *Picea koraiensis* | 129.0208 | 46.9792 | *Picea wilsonii* | 102.5208 | 36.8125 |
| *Picea koraiensis* | 129.0208 | 47.8958 | *Picea wilsonii* | 102.5625 | 30.8958 |
| *Picea koraiensis* | 129.0625 | 48.1042 | *Picea wilsonii* | 102.5625 | 35.8125 |
| *Picea koraiensis* | 129.1042 | 48.1042 | *Picea wilsonii* | 102.5625 | 36.8542 |
| *Picea koraiensis* | 129.1458 | 47.0625 | *Picea wilsonii* | 102.6458 | 35.8542 |
| *Picea koraiensis* | 129.1458 | 47.1042 | *Picea wilsonii* | 102.6875 | 35.8125 |
| *Picea koraiensis* | 129.1875 | 48.1042 | *Picea wilsonii* | 102.6875 | 36.6875 |
| *Picea koraiensis* | 129.3125 | 48.2292 | *Picea wilsonii* | 102.6875 | 36.7708 |
| *Picea koraiensis* | 129.3125 | 48.2708 | *Picea wilsonii* | 102.7292 | 36.6875 |
| *Picea koraiensis* | 129.3542 | 43.3542 | *Picea wilsonii* | 102.7708 | 36.6875 |
| *Picea koraiensis* | 129.3958 | 48.7292 | *Picea wilsonii* | 102.8125 | 31.6875 |
| *Picea koraiensis* | 129.4375 | 47.7292 | *Picea wilsonii* | 102.8958 | 36.6875 |
| *Picea koraiensis* | 129.4375 | 48.6875 | *Picea wilsonii* | 102.9375 | 34.1458 |
| *Picea koraiensis* | 129.4792 | 47.7708 | *Picea wilsonii* | 102.9375 | 35.0208 |
| *Picea koraiensis* | 129.4792 | 48.5208 | *Picea wilsonii* | 102.9792 | 32.0625 |
| *Picea koraiensis* | 129.5625 | 48.5625 | *Picea wilsonii* | 102.9792 | 33.5625 |
| *Picea koraiensis* | 129.6875 | 44.2708 | *Picea wilsonii* | 102.9792 | 33.9375 |
| *Picea koraiensis* | 129.6875 | 48.4792 | *Picea wilsonii* | 103.0625 | 32.0625 |
| *Picea koraiensis* | 129.7292 | 44.5625 | *Picea wilsonii* | 103.1458 | 34.1042 |
| *Picea koraiensis* | 129.7708 | 43.4375 | *Picea wilsonii* | 103.1875 | 31.4375 |
| *Picea koraiensis* | 129.7708 | 48.8958 | *Picea wilsonii* | 103.2292 | 33.6042 |
| *Picea koraiensis* | 129.8125 | 48.4375 | *Picea wilsonii* | 103.2292 | 34.0625 |
| *Picea koraiensis* | 130.1042 | 43.1875 | *Picea wilsonii* | 103.2708 | 34.2292 |
| *Picea koraiensis* | 130.1875 | 43.0625 | *Picea wilsonii* | 103.2708 | 35.6875 |
| *Picea koraiensis* | 130.1875 | 44.2292 | *Picea wilsonii* | 103.3125 | 32.3958 |
| *Picea koraiensis* | 130.2292 | 43.3125 | *Picea wilsonii* | 103.3958 | 35.2292 |
| *Picea koraiensis* | 130.3125 | 44.4792 | *Picea wilsonii* | 103.4375 | 34.6042 |
| *Picea koraiensis* | 130.5208 | 46.1875 | *Picea wilsonii* | 103.5208 | 31.8125 |
| *Picea meyeri* | 110.4375 | 40.4375 | *Picea wilsonii* | 103.5208 | 34.6042 |
| *Picea meyeri* | 110.7292 | 40.6875 | *Picea wilsonii* | 103.6042 | 32.6458 |
| *Picea meyeri* | 111.4375 | 37.1042 | *Picea wilsonii* | 103.6458 | 33.7708 |
| *Picea meyeri* | 111.4375 | 37.8542 | *Picea wilsonii* | 103.7708 | 34.9375 |
| *Picea meyeri* | 111.4375 | 37.8958 | *Picea wilsonii* | 103.8542 | 31.6875 |
| *Picea meyeri* | 111.4792 | 37.6458 | *Picea wilsonii* | 103.8542 | 32.7292 |
| *Picea meyeri* | 111.4792 | 37.6875 | *Picea wilsonii* | 103.8958 | 32.7708 |
| *Picea meyeri* | 111.4792 | 37.7292 | *Picea wilsonii* | 103.8958 | 33.1875 |
| *Picea meyeri* | 111.4792 | 37.7708 | *Picea wilsonii* | 103.8958 | 34.1458 |
| *Picea meyeri* | 111.4792 | 37.8542 | *Picea wilsonii* | 103.9375 | 32.7708 |
| *Picea meyeri* | 111.5625 | 37.8542 | *Picea wilsonii* | 103.9375 | 33.2292 |
| *Picea meyeri* | 111.5625 | 37.8958 | *Picea wilsonii* | 103.9375 | 35.8958 |
| *Picea meyeri* | 111.6042 | 37.8958 | *Picea wilsonii* | 104.0208 | 35.7292 |
| *Picea meyeri* | 111.6875 | 38.3542 | *Picea wilsonii* | 104.0625 | 35.7708 |
| *Picea meyeri* | 111.7292 | 38.6458 | *Picea wilsonii* | 104.0625 | 35.8125 |
| *Picea meyeri* | 111.7708 | 38.6875 | *Picea wilsonii* | 104.1042 | 33.2708 |
| *Picea meyeri* | 111.7708 | 39.0208 | *Picea wilsonii* | 104.1042 | 35.8542 |
| *Picea meyeri* | 111.7708 | 41.1042 | *Picea wilsonii* | 104.3542 | 33.5625 |
| *Picea meyeri* | 111.8125 | 38.7708 | *Picea wilsonii* | 104.3542 | 33.7708 |
| *Picea meyeri* | 111.8542 | 38.8125 | *Picea wilsonii* | 104.3958 | 33.8125 |
| *Picea meyeri* | 111.8958 | 38.7292 | *Picea wilsonii* | 104.4375 | 33.5208 |
| *Picea meyeri* | 111.9375 | 38.7292 | *Picea wilsonii* | 104.4792 | 34.6458 |
| *Picea meyeri* | 111.9375 | 38.8125 | *Picea wilsonii* | 104.5625 | 32.4375 |
| *Picea meyeri* | 111.9375 | 38.8542 | *Picea wilsonii* | 104.7292 | 32.4375 |
| *Picea meyeri* | 111.9375 | 38.8958 | *Picea wilsonii* | 104.8125 | 34.1875 |
| *Picea meyeri* | 111.9792 | 38.8542 | *Picea wilsonii* | 104.8125 | 34.5625 |
| *Picea meyeri* | 112.0208 | 38.7292 | *Picea wilsonii* | 104.8125 | 35.4792 |
| *Picea meyeri* | 112.0208 | 38.8542 | *Picea wilsonii* | 105.7292 | 33.7292 |
| *Picea meyeri* | 112.0625 | 38.9792 | *Picea wilsonii* | 105.7708 | 33.7292 |
| *Picea meyeri* | 112.1042 | 38.8125 | *Picea wilsonii* | 105.9375 | 38.7708 |
| *Picea meyeri* | 112.1042 | 38.8958 | *Picea wilsonii* | 106.1875 | 39.2708 |
| *Picea meyeri* | 112.1042 | 38.9375 | *Picea wilsonii* | 106.4792 | 35.5625 |
| *Picea meyeri* | 112.1042 | 38.9792 | *Picea wilsonii* | 106.5208 | 34.2708 |
| *Picea meyeri* | 112.3125 | 38.9792 | *Picea wilsonii* | 106.6042 | 34.1875 |
| *Picea meyeri* | 112.3125 | 40.6042 | *Picea wilsonii* | 107.8125 | 33.6042 |
| *Picea meyeri* | 113.4375 | 39.7708 | *Picea wilsonii* | 107.8125 | 34.0208 |
| *Picea meyeri* | 113.4792 | 39.0625 | *Picea wilsonii* | 107.9792 | 33.5208 |
| *Picea meyeri* | 113.5625 | 39.0208 | *Picea wilsonii* | 107.9792 | 33.7292 |
| *Picea meyeri* | 113.6042 | 39.0208 | *Picea wilsonii* | 108.2708 | 33.8542 |
| *Picea meyeri* | 113.6042 | 39.9375 | *Picea wilsonii* | 108.3125 | 33.3125 |
| *Picea meyeri* | 113.6042 | 39.9792 | *Picea wilsonii* | 108.4792 | 33.4792 |
| *Picea meyeri* | 113.6042 | 40.0625 | *Picea wilsonii* | 108.5625 | 34.0208 |
| *Picea meyeri* | 113.6458 | 39.0625 | *Picea wilsonii* | 108.6042 | 32.1042 |
| *Picea meyeri* | 113.6458 | 39.1458 | *Picea wilsonii* | 108.6458 | 31.8125 |
| *Picea meyeri* | 113.8958 | 38.9375 | *Picea wilsonii* | 108.7708 | 31.9792 |
| *Picea meyeri* | 114.8958 | 40.7708 | *Picea wilsonii* | 109.3542 | 31.9792 |
| *Picea meyeri* | 114.9375 | 39.8542 | *Picea wilsonii* | 109.5625 | 31.3958 |
| *Picea meyeri* | 114.9375 | 39.9375 | *Picea wilsonii* | 109.6458 | 31.3542 |
| *Picea meyeri* | 114.9792 | 39.8125 | *Picea wilsonii* | 109.8125 | 31.4375 |
| *Picea meyeri* | 114.9792 | 39.8542 | *Picea wilsonii* | 110.1042 | 34.4792 |
| *Picea meyeri* | 114.9792 | 39.9375 | *Picea wilsonii* | 110.2292 | 31.6458 |
| *Picea meyeri* | 115.0208 | 39.8542 | *Picea wilsonii* | 110.3125 | 31.0208 |
| *Picea meyeri* | 115.0208 | 39.8958 | *Picea wilsonii* | 110.3542 | 31.0625 |
| *Picea meyeri* | 115.0625 | 39.9375 | *Picea wilsonii* | 110.3958 | 31.6042 |
| *Picea meyeri* | 115.0625 | 39.9792 | *Picea wilsonii* | 110.4375 | 31.3958 |
| *Picea meyeri* | 115.0625 | 40.0208 | *Picea wilsonii* | 110.4792 | 31.6875 |
| *Picea meyeri* | 115.1042 | 40.4792 | *Picea wilsonii* | 110.5208 | 31.8125 |
| *Picea meyeri* | 115.1875 | 39.7708 | *Picea wilsonii* | 110.6458 | 31.4375 |
| *Picea meyeri* | 115.2292 | 39.8125 | *Picea wilsonii* | 110.6458 | 31.4792 |
| *Picea meyeri* | 115.2292 | 40.0625 | *Picea wilsonii* | 110.6875 | 31.7292 |
| *Picea meyeri* | 115.2708 | 39.3125 | *Picea wilsonii* | 110.7292 | 32.1458 |
| *Picea meyeri* | 115.3542 | 39.3542 | *Picea wilsonii* | 110.7708 | 31.3125 |
| *Picea meyeri* | 115.4375 | 39.3542 | *Picea wilsonii* | 110.9375 | 30.6042 |
| *Picea meyeri* | 115.4792 | 40.0625 | *Picea wilsonii* | 111.2292 | 40.8958 |
| *Picea meyeri* | 115.6042 | 39.8542 | *Picea wilsonii* | 111.2708 | 30.9792 |
| *Picea meyeri* | 115.8125 | 42.8542 | *Picea wilsonii* | 111.4375 | 38.4375 |
| *Picea meyeri* | 116.3125 | 43.2292 | *Picea wilsonii* | 111.4792 | 37.8542 |
| *Picea meyeri* | 116.5208 | 42.8958 | *Picea wilsonii* | 111.8958 | 36.6042 |
| *Picea meyeri* | 116.6458 | 42.5208 | *Picea wilsonii* | 111.9792 | 35.3958 |
| *Picea meyeri* | 116.8542 | 42.8125 | *Picea wilsonii* | 111.9792 | 38.6875 |
| *Picea meyeri* | 116.8958 | 42.4375 | *Picea wilsonii* | 112.1042 | 36.6042 |
| *Picea meyeri* | 116.9792 | 42.4375 | *Picea wilsonii* | 112.1042 | 38.8542 |
| *Picea meyeri* | 117.2292 | 42.3958 | *Picea wilsonii* | 113.1458 | 30.6458 |
| *Picea meyeri* | 117.3542 | 42.4375 | *Picea wilsonii* | 113.6458 | 38.7292 |
| *Picea meyeri* | 117.4792 | 40.6042 | *Picea wilsonii* | 113.7292 | 39.6875 |
| *Picea meyeri* | 118.3125 | 43.9375 | *Picea wilsonii* | 114.3125 | 30.5625 |
| *Picea meyeri* | 118.4375 | 41.3958 | *Picea wilsonii* | 115.5208 | 40.0625 |
| *Picea mongolica* | 116.3125 | 43.2292 | *Picea wilsonii* | 115.7708 | 40.4792 |
| *Picea mongolica* | 116.4792 | 42.8958 | *Picea wilsonii* | 116.1042 | 41.3125 |
| *Picea mongolica* | 116.5208 | 42.8958 | *Picea wilsonii* | 117.3542 | 40.6042 |
| *Picea mongolica* | 116.5208 | 43.5625 | *Picea wilsonii* | 117.4792 | 40.8958 |
| *Picea mongolica* | 116.6042 | 42.6875 | *Picea wilsonii* | 118.1458 | 41.4375 |
| *Picea mongolica* | 116.6458 | 42.5208 | *Picea wilsonii* | 118.3125 | 41.6458 |

Table S9 Deposition number of spruce materials

| Population | Species | Voucher | BioSample Accession |
| --- | --- | --- | --- |
| Ret | *Picea retroflexa* | RG302022 | SAMN35675616-35675603 |
| Mon1 | *Picea mongolica* | MG092022 | SAMN35675582-35675569 |
| Mon2 | *Picea mongolica* | MG272022 | SAMN35675568-35675554 |
| Kor2 | *Picea koraiensis* | K072022 | SAMN35675553-35675541 |
| Kor1 | *Picea koraiensis* | K012022 | SAMN35675540-35675526 |
| Mey4 | *Picea meyeri* | G412022 | SAMN35675520-35675506 |
| Mey3 | *Picea meyeri* | G402022 | SAMN35675505-35675491 |
| Mey2 | *Picea meyeri* | G242022 | SAMN35675490-35675476 |
| Mey1 | *Picea meyeri* | G232022 | SAMN35675475-35675461 |
| Cra | *Picea crassifolia* | CG132022 | SAMN35675460-35675449 |
| Asp | *Picea asperata* | AG162022 | SAMN35675448-35675432 |
| Wil | *Picea wilsonii* | WG292022 | SAMN30656382-30656368 |
| Pun | *Picea pungens* | PG392022 | SAMN30656367-30656363 |
| Neo2 | *Picea neoveitchii* | NG332022 | SAMN30656362-30656353 |
| Neo1 | *Picea neoveitchii* | NG322022 | SAMN30656352-30656348 |
| Jez | *Picea jezoensis* | JG142022 | SAMN30656342-30656338 |
